# Barriers and facilitators to consuming pulses: a qualitative exploration including effects of trying recipes at home

**DOI:** 10.1017/jns.2023.119

**Published:** 2024-01-31

**Authors:** B. Whittall, S. M. Warwick, M. Jackson, K. M. Appleton

**Affiliations:** Research Centre for Behaviour Change, Department of Psychology, Bournemouth University, Poole, UK

**Keywords:** awareness, experience, knowledge, sustainability, thematic analysis

## Abstract

Pulses are a healthy, sustainable, low cost food, but consumption levels are low for a variety of reasons, including practical and cooking concerns. This work aimed to explore barriers and facilitators towards pulse consumption and increasing consumption, and the potential value of cooking suggestions and recipes for changing these perceptions. Two qualitative studies were undertaken. In Study 1, 21 participants (10 males, 11 females, of a range of ages, cooking responsibilities, and experiences with pulses) were interviewed both before and after receiving cooking suggestions and recipes. In Study 2, 12 participants (2 males, 10 females, as above) were interviewed once after trying recipes. Interviews were audio-recorded, transcribed, and analysed using thematic analysis. Seven themes described barriers and facilitators towards pulse consumption: ‘Enjoyment and Sensory properties’; ‘Benefits and Recommendations’; ‘Practical Concerns’; ‘Cooking Concerns’; ‘Compatibility with current diet’; ‘Personal Influences’; and ‘External Influences’. Some similar themes also referred to increasing consumption: ‘Willingness’; ‘Awareness, Knowledge of Benefits’; ‘Knowledge of Cooking and Practical Concerns’; and ‘Compatibility with current diet’. Cooking suggestions and recipe use resulted in themes on ‘Awareness’; ‘Willingness, Trying New Things’; ‘Small Changes’; and facilitators associated with ‘Enjoyment, Sensory Properties, Practical Concerns, Benefits’ and ‘Knowledge, Cooking Ideas and Confidence, Incorporation, Cooking Solutions’. Barriers related to ‘Risk and Preconceptions’; ‘Awareness, but’ inaction and additional considerations were also found. Our findings demonstrate a positive role for pulse consumption for increased experience, familiarity, and confidence with preparing, cooking, and consuming these healthy and sustainable foods.

## Introduction

Pulses are defined as the dry (non-oil) edible seeds of plants from the botanical family *Leguminosae* or *Favaceae,* to include dry beans, dry peas, chickpeas, and lentils^(1)^. They are high in protein, complex carbohydrates, including resistant starch, soluble and insoluble fibre, low in saturated fat, high in many micronutrients, including folate, iron, magnesium, potassium, choline, zinc, selenium, phosphorus, and thiamine^(2,3)^, and contain many non-nutritive bioactive compounds, including phytochemicals and flavonoids, that offer anti-oxidant, anti-cancer, and protective activities^(2–4)^. Pulse consumption is associated with dietary profiles that are higher in protein, fibre, and various micronutrients, than are found for non-consumers^(5–7)^, and these nutrient profiles can confer considerable health benefits. Pulse consumption is associated with improved blood glucose control and insulin sensitivity, improved blood lipid and cholesterol profiles, and with increased satiation and reduced energy intake^(2–4,8)^. Beneficial impacts are reported for bodyweight and obesity^(2,7–10)^, blood pressure, cardiovascular disease risk and metabolic syndrome^(2,3,7,9–11)^, diabetes risk^(2,3,12)^, and some cancers^(4)^. Health benefits are also likely gained not only from the nutritional properties of pulses but also from the displacement of less healthy foods within the diet, such as red meat, processed meats, and less complex carbohydrates^(3,4,11)^.

Considering energy and nutrient density, pulses, furthermore, are inexpensive and rank high in value for money^(13)^. Pulses are also considered a food of low environmental impact^(14–18)^. Greenhouse gas emissions are reported at 0.8 – 3.5 kgCO^2^/kg in the UK and Ireland for UK and EU grown beans and pulses compared to 5 – 8 kgCO^2^/kg for meat from poultry and pigs, 33 – 36 kgCO^2^/kg for other meats and offal, and 64 – 69 kgCO^2^/kg for mutton, goat and beef^(14,15)^. Freshwater withdrawals (scarcity-weighted) are comparable for pulses, eggs, and poultry, at approximately half of that required for grains and beef, while requirements for pig meat, sheep meat, and dairy are higher^(15)^. Land use is also comparable for pulses, pig meat, cheese, and eggs, while land for beef and sheep herds can be 8 – 10 times this^(16)^. The Eat Lancet Planetary Health diet recommends consumption of at least 75g/d legumes, which includes pulses^(17,18)^.

However, while healthy, inexpensive and sustainable, pulse consumption is low^(3,9,10,19)^. Consumption of legumes and nuts in the UK was most recently reported as 31g/d^(19)^ and in Europe ranges between 9g/d and 26g/d, to result in an average consumption of 15g/d^(19)^. Similar low levels of consumption are also reported in the US and Canada^(3,9,10)^.

Reasons for not consuming pulses include disliking of the taste and/or texture^(20–22)^, low knowledge of the value of pulses for health^(20,23)^, lack of experience or familiarity with pulses as they are not a traditional part of the diet^(20,24)^, the perceived effort, time, and inconvenience of preparing and cooking pulses^(3,21,23–25)^, lack of practical cooking knowledge and confidence^(20–23,25)^, gastro-intestinal effects such as flatulence and intestinal discomfort^(3,20,23,24)^, and negative culinary image, as pulses are perceived as a ‘poor man’s food’^(3,20,22,25)^, a poor substitute for meat^(25)^, or as a food only for vegetarians^(21)^.

Solutions to many of these barriers can be suggested. As examples, knowledge of health and nutritional benefits can be increased through information and education^(21,22,24)^, although knowledge is often described as insufficient alone to change behaviour^(21.26)^. It has been suggested that the practical concerns around pulses can be reduced through the use of pre-prepared and canned pulses or through simple preparation techniques, such as batch cooking and freezing^(22,24,27,28)^. Similarly, cooking concerns may be reduced through the use of simple cooking suggestions, such as incorporating pulses into existing dishes like soups and stews^(22,25,27,28)^, and the provision of recipes for tasty meals composed of pulses^(21,25,28)^. Tasty solutions may improve liking, increase familiarity and, while the incorporation of pulses into well-known and familiar meals may aid acceptance, use of recipes from cultures that traditionally use pulses, such as Mexico, the Middle East, and India have also been suggested to provide opportunities for taste experiences^(21,27)^. While these solutions can be suggested, however, no study of which we are aware has tested the value of these suggestions for improving pulse consumption.

This work aimed to explore the potential value of these practical solutions for improving pulse consumption. First, barriers and facilitators towards consuming pulses were explored. Second, barriers and facilitators towards increasing pulse consumption were explored. Third, barriers and facilitators towards consuming pulses were again explored following requests to try some pulse-based recipes at home. Two studies were undertaken using qualitative methodology. In Study 1, participants were interviewed to explore barriers and facilitators towards consuming pulses and towards increasing pulse consumption, and three weeks later, they were interviewed again to investigate any changes to these barriers and facilitators following a request to try some recipes at home. In Study 2, participants were interviewed after trying some recipes including pulses at home. Study 2 was undertaken to supplement Study 1, as several participants in Study 1 did not try any recipes. These participants provided valuable data on why they did not try the recipes, but could contribute no data on the effects of trying recipes. The studies were exploratory; thus, there were no hypotheses to be tested.

## Methods

### Participants

Participants were required to be aged 18 years or over, identify as British, be able to provide informed consent and undertake all aspects of the study. No additional inclusion criteria were used to gain a wide variety of opinions from a variety of participants, to include participants from both genders, with a range of ages, living circumstances, cooking responsibilities, dietary choices, and a range of experiences with pulses. Recruitment focussed on young adults, as a population group who may be more amenable to dietary change, and where benefits may accrue over the long term^(29)^, although volunteers of any age were included. All individuals who volunteered for the research took part. Recruitment was undertaken over the Dorset area in the UK using social media, local public advertisements, and word-of mouth, for ‘*Studies into Current Diets’*. Both studies were conducted according to the guidelines laid down in the Declaration of Helsinki, and all procedures involving human participants were approved by the Research Ethics Committee of Bournemouth University, UK, prior to commencement (Study 1: ID: 34632; Study 2: ID: 34658). Written informed consent was obtained from all participants.

### Data collection

Both studies took a qualitative approach, using individual interviews. Interviews were considered an appropriate methodology to gain detailed, rich, and personal responses from participants^(30)^ and were considered a practical method at the time the research was conducted. Each interview started with an explanation of the study and study procedures, and confirmation of consent.

In Study 1, questions on current diet were then asked, to establish rapport and familiarise participants with the interview process. Following this, participants were queried on their pulse consumption, reasons for consuming or not consuming pulses, willingness to increase pulse consumption, and how this may be facilitated or hindered. All questions were open and broad to elicit a range of responses and were asked in an open, accepting, and non-judgemental manner^(30)^. Questions were supplemented with a 4 minute information video, provided to participants after some initial questions on knowledge and use. This video, by a qualified dietitian, gave some technical knowledge of pulses, several benefits that can be gained from eating pulses, and included three dishes that incorporated pulses – a tomato soup, a chilli-type main dish, and a chocolate brownie. The video was provided towards to the start of the interview to ensure the rest of the interview was conducted following a clear understanding of the topic of interest and was chosen to provide some information on the benefits of pulse consumption and to highlight the ease with which pulses could be consumed. At the end of the interview, participants were provided with the recipes for the dishes featured, were given a voucher to procure all ingredients, and were asked to try at least one of them. Participants then returned three weeks after the first interview to undertake a second interview. This second interview asked for recipe use, any changes that participants had made to their pulse consumption, reasons for these changes, and any other barriers or facilitators to making changes.

In Study 2, only the second interview was undertaken. On recruitment into the study, participants were provided with the information from the video and were then provided with three recipes (lentil and mushroom meatballs, peanut butter and chickpea cookies and white bean vanilla cake), asked to try at least one of them and given a voucher to procure the necessary ingredients.

All interviews followed an interview schedule that was piloted prior to use. The interview schedules for both interviews are given in Table 1. Interviews were conducted and recorded over Zoom, then transcribed, from February to May, 2021, when COVID-19-related restrictions on activities were in place across the UK. All participants in Study 1 also took part in an earlier interview on sustainable diets with the same researcher.

**Table 1.** Interview schedules

**Interview 1**	
	**Topic: Current Diet**
1.	Can you tell me about your current diet?
2.	Why do you think you have adopted your diet?
3.	Who normally prepares your meals?
	If you do, do you also cook for others?
	Do others have an impact on what you cook?
	If someone else, to what extent can you impact what they make?
4.	Are there any changes you would like to make to your diet?
	If so, what are they?
5.	Are there any barriers to changing your diet?
6.	Would there be any benefits to changing your diet?
7.	Is there anything else you would like to talk about regarding your current diet or the beliefs you have about your diet?
	**Topic: Pulses**
8.	Can you tell me about what you think pulses are?
9.	What, if any, are your experiences of pulses?
	What do you think of them?
	What do you do with them?
	**Information video**
10.	Has the video given you a better understanding of pulses?
	Was there anything you found interesting?
	Was there anything you didn’t know before?
11.	Do you think pulses would be beneficial for your diet?
12.	What do you think are good, and what do you think are bad about them?
13.	Thinking about the benefits of pulses, to what extent do you think you would try to incorporate pulses into your diet?
14.	How much in the next 3 weeks do you think you’ll try to incorporate more pulses into your diet?
15.	Would there be any barriers to adding more pulses into your diet?
16.	Anything that might help?
17.	Would you do more of your own research into this area?
18.	Is there anything else you would like to talk about in relation to pulses?
**Interview 2**	
	**Topic 1: Current diet**
1.	Can you tell me about your diet and beliefs before the first interview/before your inclusion in the study, and how they differ now, if they do?
2.	To what extent has your diet changed over the last few weeks?
	Have there been any challenges?
	Have there been any positives?
3.	Is there anything else you would like to talk about in relation to your diet?
	**Topic: Pulses**
4.	Can you tell me about your beliefs and understanding about pulses before the first interview?/before your inclusion in the study?
5	Did you eat pulses then?
	Why/Why not?
6.	Have you tried to adopt pulses into your diet?
	Why/Why not?
7.	Did you try any of the recipes?
	Why did you try that one/those ones?
	Why didn’t you try any/other recipes?
8.	After trying the recipes, what are your beliefs about pulses now?
	How has that changed?
	Would you try more recipes?
9.	Would you use or try any pulse-based recipes in the future?
	Why? Why not?
10.	What are the barriers to adopting pulses into your diet, if any?
11.	What might encourage you?
12.	Is there anything else you would like to talk about?

### Data analysis

The analysis plan was pre-specified in advance of data collection. Interview transcripts were analysed using thematic analysis, following the six steps of Braun & Clarke^(31)^: (1) read and familiarise self with the script; (2) identify initial codes within the data; (3) collate codes and identify possible themes; (4) review and check if themes fit across all datasets and create a thematic map of the analysis to address the main topics identified; (5) define and name themes; and (6) present results. Thematic analysis was considered suitable considering the topic of the interviews as non-sensitive and a topic that participants were willing to discuss openly^(31)^. All interviews and transcripts were completed by one researcher (Study 1: BW; Study 2: MJ). All transcripts were then read and coded by these researchers and a further independent researcher (SMW or KMA), and all codes were subsequently agreed. All codes were data-derived; no pre-specified coding or theoretical framework was applied, to encourage the use of unconstrained codes^(32)^. Interviews and analyses for Study 1 were undertaken alternately to allow an assessment of the number of new codes arising per interview, and interviews for Study 1 were stopped when no new codes were found in two consecutive analysed transcripts as a marker of data saturation^(33)^. Study 2 was undertaken shortly after the second interview for Study 1 when the number of participants who had tried recipes was known. All interviews (Study 1, interview 1; Study 1, interview 2; Study 2, interview 2) were also analysed and coded over the same time period. The agreement between coders was 91%. Once all codes were confirmed, codes were then discussed by all researchers and collated based on underlying topic to result in themes^(30)^. Transcripts were typed, but no specific software was used during analysis, the researchers preferring handwritten notes and ‘post-it’ notes. Finally, all researchers checked and confirmed the reporting of all themes as provided here.

### Researchers and reflexivity

All four researchers were female. Two researchers have a background in Health Psychology, one has a background in diet and nutrition and one has a mixture of both. All of the researchers have an interest in and practice healthy eating and have interests in sustainability. The work was undertaken as a result of the interests of the two main researchers (BW, KMA) with genuine interests in gaining as much useful knowledge in this area as possible. The validity of the work was aided by the additional researchers, so reducing potential biases due to prior knowledge, but the backgrounds and interests of all researchers may have influenced the identification of themes and interpretation of the data^(34)^. All researchers were also aware of a current social desirability bias towards sustainability in the UK and sought to ensure open and genuine responses from participants and analyses, but social desirability remains a threat in this topic area^(35,36)^.

## Results

### Participants

For Study 1, 21 participants took part: 11 females, 10 males. The majority of participants were aged 20–25 years, but four participants were aged 26–35 years, and three participants were aged 50–55 years. Three participants lived with others and usually cooked for the household, three participants lived with others and shared the cooking, four participants lived with others and sometimes cooked, five participants lived alone or with others and cooked for themselves, five participants lived with others and were usually cooked for, and one participant lived with others and had recently taken over most of the cooking for the family. In relation to dietary choices, four participants mentioned an active lifestyle that warranted high food intakes, two participants specifically mentioned enjoying cooking and consuming from a variety of cuisines, three participants described their diets as unhealthy, three participants reported dietary restrictions, and six participants specifically reported trying to change their diets to consume more healthy or sustainable foods. Prior to the first interview, familiarity with pulses was varied, ranging from habitual and regular use once or twice a week to no current consumption. Knowledge regarding pulses was varied, ranging from no knowledge at all to considerable knowledge. Prior to the study, all participants had taken part in an earlier interview on sustainable diets^(37)^. Prior to the second interview, all participants had undertaken the first interview, but changes to diets in the intervening period were varied, as were uses of the recipes provided. Some participants tried one or several of the recipes, some participants didn’t try the recipes provided, but did try different recipes instead, and some participants didn’t try either the recipes provided or other recipes, and made no changes to their diets. Brief characteristics of each participant are given in Table 2.

**Table 2.** Brief characteristics of participants in Study 1 (*N* = 21)

	Gender	Age	Living situation	Cooking responsibilities	Prior experience of pulses	Experience gained
*B1*	Male	23	Lives with parents	Take turns to cook	Eats them, if already included	Tried 1 recipe – positive experience
*B2*	Male	23	Lives with family	Is usually cooked for	Eat beans/peas	Tried 1 recipe – positive experience
*B3*	Female	24	Lives with partner	Sometimes cooks	Not much experience until recently	No changes. Didn’t try recipes.
*B4*	Male	27	Lives with partner	Is usually cooked for	Not much experience, but has eaten them	Added more pulses. Didn’t try recipes.
*B5*	Male	22	Lives with parents	Sometimes cooks	Not much experience, but has eaten them	Tried 1 recipe – positive experience
*B6*	Male	23	Lives with friends	Usually cooks for himself	Uses them in foods	Added more pulses. Tried other recipes.
*B7*	Female	24	Lives with partner	Sometimes cooks	Likes them	Added more pulses. Tried other recipes.
*B8*	Male	22	Lives with parents	Cooks for himself	Eats them, if already included	Some changes. Didn’t try recipes.
*B9*	Male	22	Lives with friends	Cooks for himself	Eats them, but not often	Tried 1 recipe – positive experience
*B10*	Female	30	Lives alone	Cooks for herself	Loves them	Tried 1 recipe – positive experience
*B11*	Female	57	Lives with family	Usually cooks	Eats them, if already included	Tried 1 recipe – positive experience
*B12*	Female	23	Lives with housemate	Take turns to cook	Uses them in foods	Tried many recipes – positive experiences
*B13*	Female	22	Lives with housemate	Take turns to cook	Eats them a lot	Tried many recipes – positive experiences
*B14*	Female	26	Lives with partner	Usually cooks	Eats them a lot	Tried 1 recipe – positive experience
*B15*	Male	58	Lives with family	Recently started cooking for family	No experience	Tried many recipes – positive experiences
*B16*	Female	23	Lives with parents	Usually cooks	Eats them a lot	Added more pulses. Didn’t try recipes.
*B17*	Female	53	Lives with family	Is usually cooked for	Eats them a lot	Added more pulses. Didn’t try recipes.
*B18*	Male	31	Lives with housemate	Cooks for himself	Eats them, but not often	Added more pulses. Didn’t try recipes.
*B19*	Male	23	Lives with family	Is cooked for and cooks for himself	Eats beans/peas	No changes. Didn’t try recipes.
*B20*	Female	23	Lives with parents	Is usually cooked for	Used to eat them	Tried many recipes – positive experiences
*B21*	Female	23	Lives with parents	Is usually cooked for	Uses them in foods	No changes. Didn’t try recipes.

For Study 2, 12 participants took part: 10 females, 2 males. Six participants were aged 18–25 years, three participants were aged 40–45 years, and three participants were aged 60–65 years. Two participants lived by themselves and usually cooked for themselves, two participants lived with others and usually cooked for themselves, six participants lived with others and usually cooked with or for others, and two participants lived with others and were usually cooked for. Prior to the study, familiarity with pulses was varied, ranging from habitual and regular use, once or twice a week, to no current consumption. Prior to the interview, all participants had tried at least one recipe, either one of those provided or one of their own, but experiences of the recipes were varied. The majority of participants reported a positive experience, but one participant reported a neutral experience and one a negative experience. Brief characteristics of each participant are given in Table 3.

**Table 3.** Brief characteristics of participants in Study 2 (*N* = 12)

	Gender	Age	Living situation	Cooking responsibilities	Prior experience of pulses	Experience gained
*M1*	Female	18	Lives with others	Is usually cooked for	Eats them, but not often	Tried all 3 recipes - positive experiences
*M2*	Female	25	Lives with others	Cooks for others	Eats them, but not often	Tried 1 recipe - positive experience
*M3*	Female	24	Lives by herself	Cooks for herself	Eats them a lot	Tried 1 recipe - positive experience
*M4*	Female	20	Lives with others	Cooks for others	Eats them a lot	Made a different recipe - positive experience
*M5*	Female	43	Lives with others	Cooks for others	Eats them, but not often	Made a different recipe - positive experience
*M6*	Female	40	Lives with others	Cooks for others	Eats them a lot	Made a different recipe - positive experience
*M7*	Female	61	Lives with others	Cooks for others	Eats them, but not often	Tried 1 recipe - negative experience
*M8*	Female	64	Lives with others	Cooks for herself	Did not eat them before the study	Tried 1 recipe - positive experience
*M9*	Female	18	Lives with others	Cooks for herself	Did not eat them before the study	Tried 1 recipe - positive experience
*M10*	Female	18	Lives with others	Is usually cooked for	Eats baked beans	Tried 1 recipe - neutral experience
*M11*	Male	42	Lives with others	Cooks with others	Eats them, but in only a few dishes	Made a different recipe - positive experience
*M12*	Male	60	Lives by himself	Cooks for himself	Eats them, but in only a few dishes	Made a different recipe - positive experience

### Themes

**Fig. 1.**
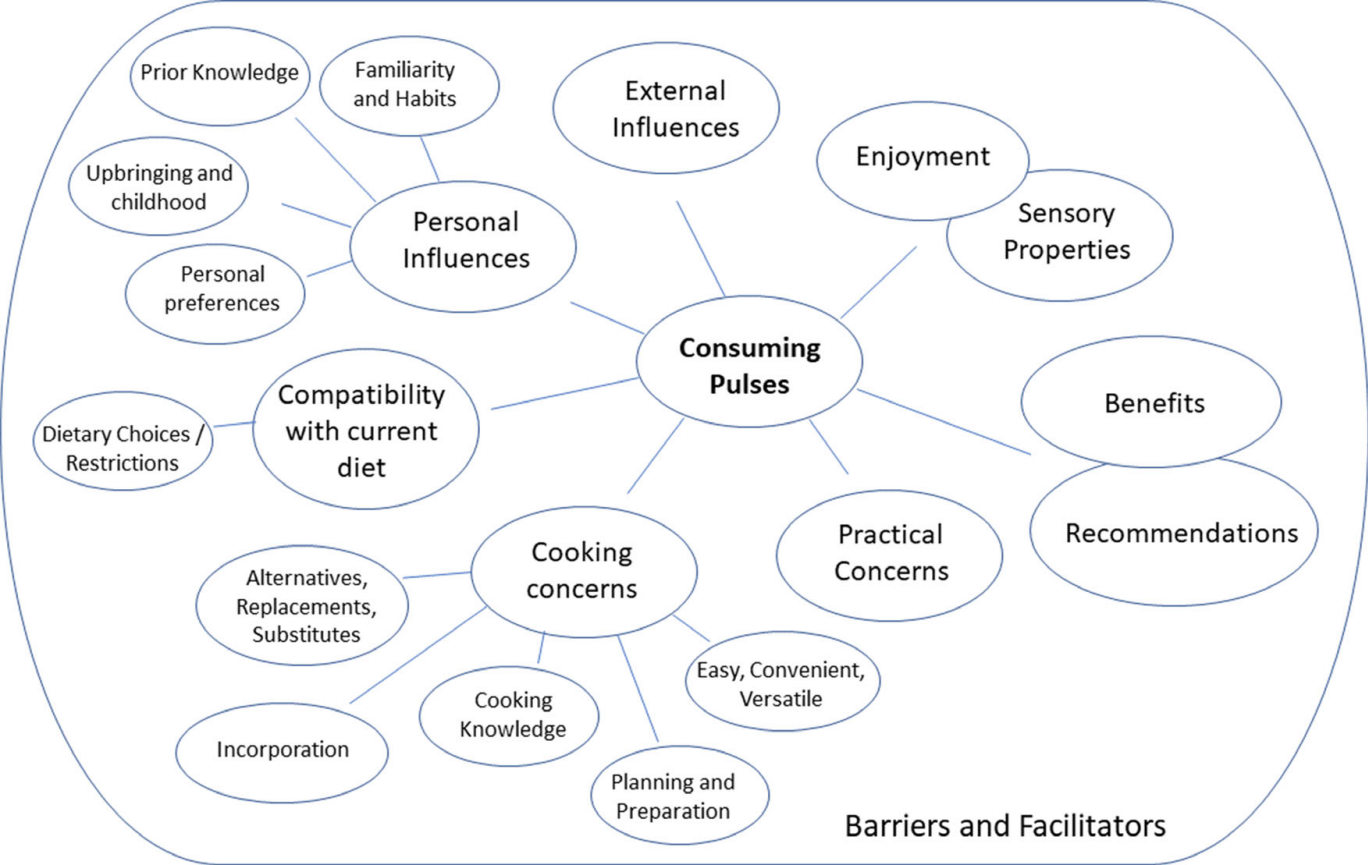
Theme map to show the themes related to barriers and facilitators towards consuming pulses.

**Fig. 2.**
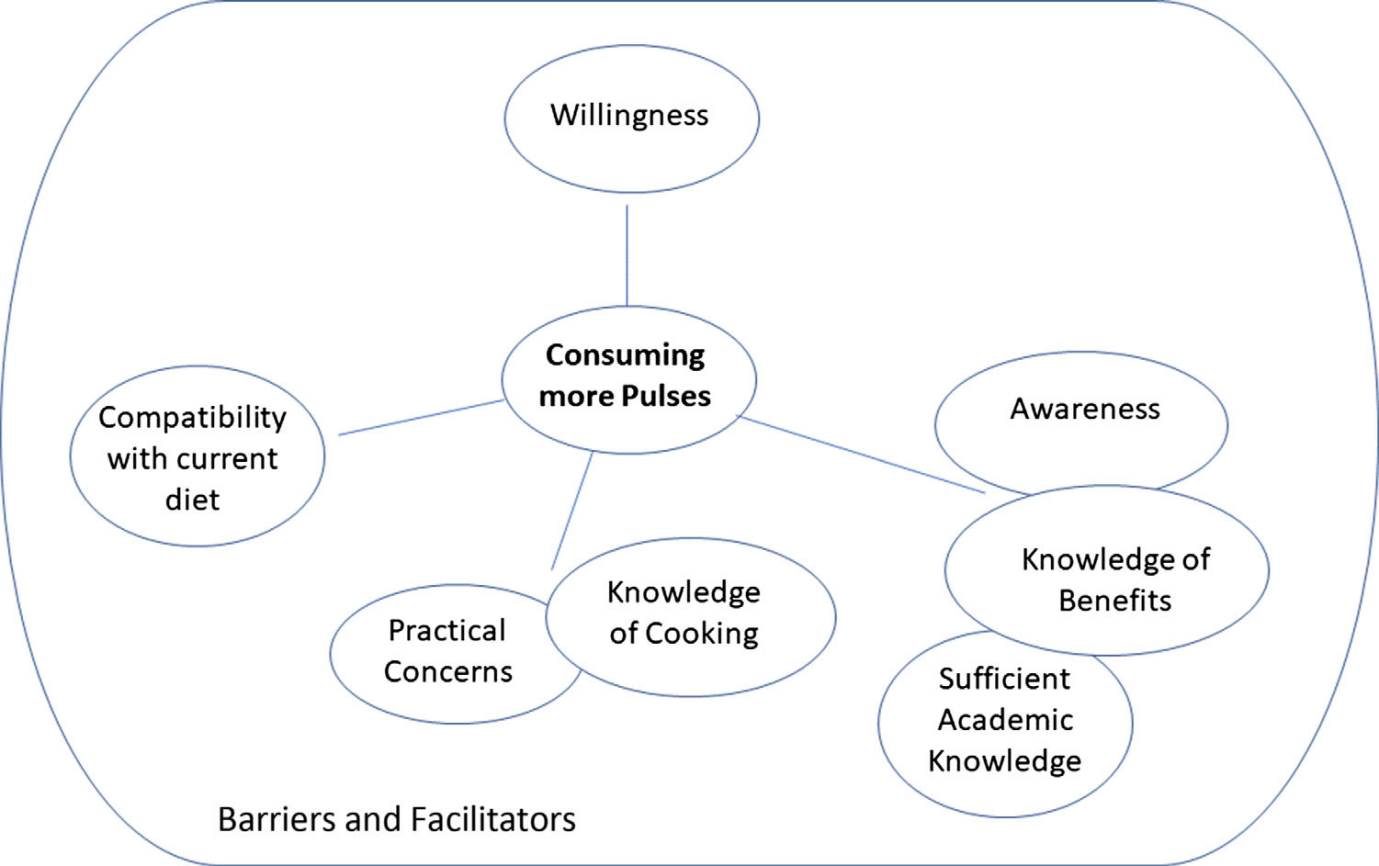
Theme map to show the themes related to barriers and facilitators towards consuming more pulses.

**Fig. 3.**
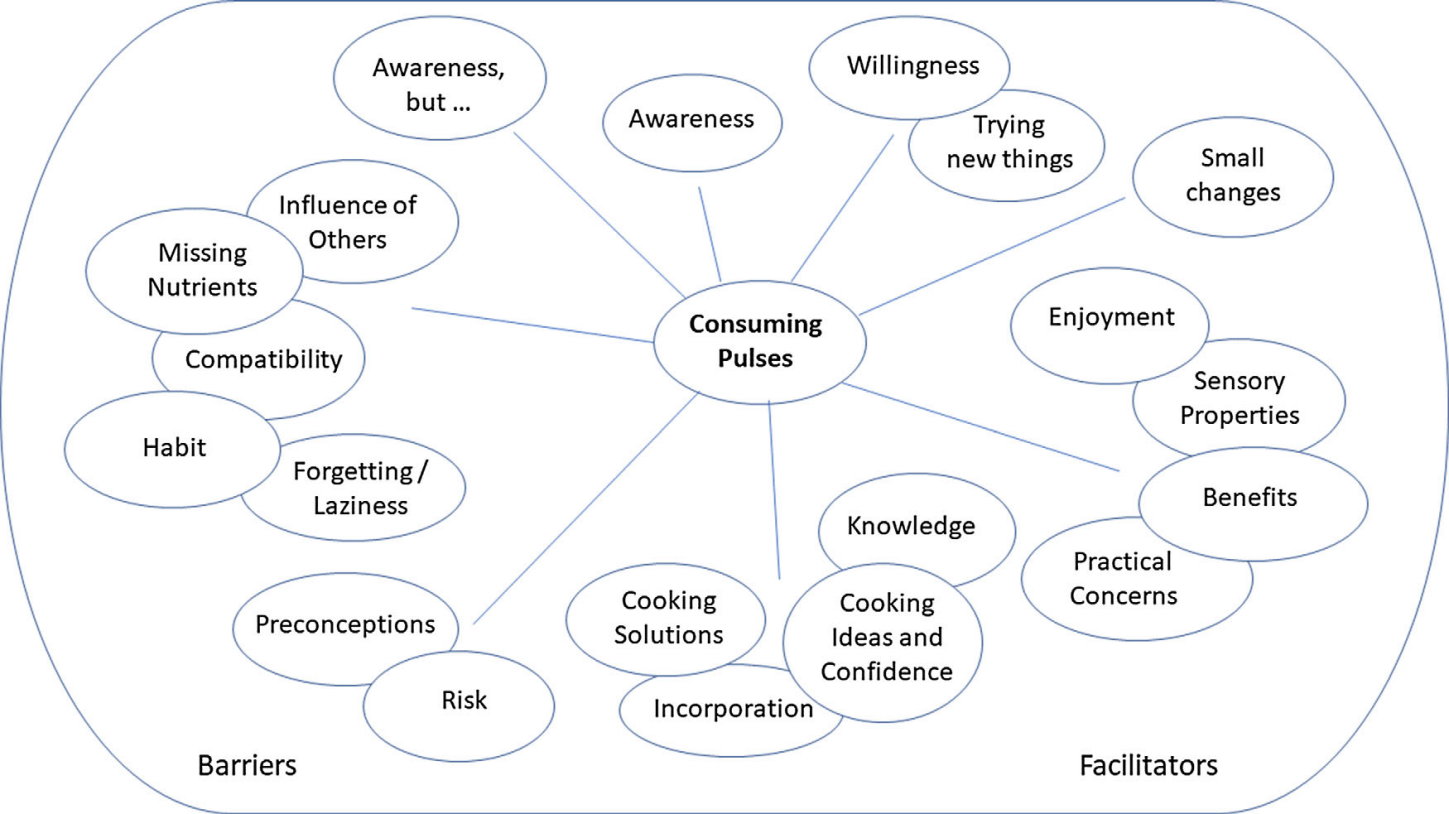
Theme map to show the themes related to barriers and facilitators towards consuming pulses following recipe provision.

Interviews from both studies were analysed and coded together. Results are presented for the three research aims: barriers and facilitators towards consuming pulses; barriers and facilitators towards increasing pulse consumption; and barriers and facilitators following requests to try some pulse-based recipes at home; thus, material relevant to each aim is included per section regardless of the interview or interview question that elicited it. Theme maps for each research aim are given in Figs. 1–3. All themes are presented below. Integral sub-themes and quotes per sub-theme are given in the Supplementary Materials. No additional codes were found in Study 2, compared to those found in Study 1, thus codes and quotes from both studies are presented together.

#### Barriers and facilitators towards consuming pulses

Several barriers and facilitators towards pulse consumption were found, classified into seven themes: ‘Enjoyment and Sensory properties’; ‘Benefits and Recommendations’; ‘Practical Concerns’; ‘Cooking Concerns’; ‘Compatibility with current diet’; ‘Personal Influences’; and ‘External Influences’.

*Enjoyment and sensory properties.* Participants referred to the enjoyment they gained from eating pulses, or the lack of enjoyment, and for many participants this enjoyment or lack of enjoyment was linked to the sensory properties of pulses, specifically their taste and texture. Tastes were described as ‘*good*’, ‘*bland’*, ‘*tasteless’,* or simply ‘*not good’*. Concerns over taste and texture were also reported to outweigh other characteristics of potential importance to consumption or non-consumption, such as health benefits.

*Benefits and recommendations*. Several benefits of consuming pulses were recognised. These were related to the nutritional composition of pulses – their protein and fibre content primarily, but mention of carbohydrates and other components of benefit for health, such as micronutrients, were also given. Benefits for a number of health conditions were specifically mentioned, as offered in the information provided, and general perceptions of increased health and increased well-being as a result of being healthy and consuming ‘*natural*’ foods were also given. Some participants explicitly commented on the recommended quantities for consumption that were given and the small portions required to gain benefits. Alongside health benefits, the nutritional composition of pulses was also recognised to add bulk and increase feelings of fullness or satisfaction from a meal. Some discomfort as a result of these components was also described in the form of possible flatulence, and some safety concerns were given as a result of potential toxins.

*Practical concerns*. Practical issues focused on availability, cost, storage, and waste. The availability of pulses, or of many different pulses, was offered to both facilitate and hinder consumption, mainly based on good or poor pulse availability at the store where participants did most of their grocery shopping. Low cost and easy storage were offered solely as facilitators of consumption. Waste was again described as a facilitator and barrier due to low perishability or inexperience during preparation and cooking, respectively.

*Cooking concerns: easy, convenient, versatile; planning and preparation; cooking knowledge; incorporation; alternatives/replacements/substitutes.* In relation to cooking, pulses were recognised as convenient, easy to use, and versatile, and at the same time as inconvenient and effortful if planning and preparation were required due to the use of dried pulses. Some participants explicitly mentioned the need for more knowledge on how to prepare pulses, and on the different ways in which they could be prepared and cooked, e.g. through the use of recipes. Convenience and ease increased with the recognition that pulses could be easily incorporated into existing dishes and could act as a substitute for other components of a meal. Participants also recognised additional benefits of incorporation in that bland tastes could be masked, textures could be used to benefit and add bulk to dishes, and that health benefits could be gained without any necessary knowledge or disadvantage. Some participants did voice concerns that pulses could not substitute for some meal or recipe components, particularly meat and vegetables, or that the substitution might be dissatisfying.

*Compatibility with current diet, including dietary choices/restrictions*. Related to cooking and preparation, there were also some concerns that pulse consumption might or would not be compatible with a current diet, or that there might be some aspects of their current diet that some participants would not be willing to give up for pulses. Some dietary choices, such as vegetarianism, and some dietary restrictions, such as gluten-free, were found to benefit from substitution with pulses.

*Personal influences: familiarity and habits; prior knowledge; upbringing and childhood experiences; personal preferences.* Familiarity, habit, and prior knowledge were generally reported where greater familiarity, prior experience, and knowledge of pulses was facilitative, and lesser was a barrier. Other personal influences were upbringing and childhood experiences, and personal preferences, with recognition that these could both facilitate and hinder pulse consumption. Facilitative childhood experiences were closely linked to increased familiarity and habitual use, while the reverse was the case for experiences that typically hindered current consumption. Preferences referred mainly to liking and disliking.

*External influences*. Finally, external influences were also recognised to influence consumption. These external influences included partners, parents, other family members, other individuals living in the same house or that may be cooked for, and individuals on social media. All these individuals were reported to potentially encourage or discourage pulse consumption dependent of habits, preferences, and needs.

#### Barriers and facilitators towards consuming more pulses

Four themes were related to consuming more pulses: ‘Willingness’; ‘Awareness, Knowledge of Benefits’; ‘Knowledge of Cooking and Practical Concerns’; and ‘Compatibility with current diet’.

*Willingness*. Participants felt that they would be willing to eat more pulses. For some participants, this was something they ‘*might try’* or ‘*would potentially attempt’*; an activity described with hesitancy and a lack of commitment, while other participants were more positive. Some participants were very positive and suggested using this opportunity to also make bigger changes to their diets.

*Awareness, knowledge of benefits, including sufficient academic knowledge*. Participants reported being unaware of the variety of benefits that could be gained from pulse consumption when informed of these and suggested that this information could encourage increased consumption. Participants were interested in gaining knowledge on the benefits of pulse consumption for their health, for their diet, or for the environment and thought this knowledge may also be useful for others. Knowledge of recommendations was considered beneficial, as were reminders of pulses and previous consumption, and/or an awareness of current levels of low consumption. Some participants, however, also felt that they had sufficient knowledge, or sufficient knowledge in a theoretical or academic sense.

*Knowledge of cooking and practical concerns*. Alongside, or instead of, this academic knowledge, participants also wanted more practical knowledge. They wanted to know how pulses could be used, how they could be incorporated into the diet, particularly to create a balanced diet, or balanced dishes, how they could be made tasty, e.g. through recipes, and suggested a need for greater cooking confidence. Demonstration of the incorporation of pulses into a few simple dishes was suggested to help by showing the ease with which these dishes could be created. Some participants mentioned a willingness to try, but also included a caveat that some of these practical concerns may be barriers, e.g. *‘I’ll give it a go, provided it’s easy’*. Participants also recognised that changes in cost, taste, and texture may also provide barriers, e.g. *‘I’ll give it a go, provided the taste doesn’t change’*. Social and living circumstances were also given as potential practical barriers, such that change may be possible dependent on acceptance or the actions of other family members, or dependent on a change in living situation.

*Compatibility with current diet*. Some participants, however, also felt that they ate enough pulses already, that they had no room in their current diet for pulses or more pulses, or that pulse consumption was simply unsuitable or incompatible with the foods or diet that they currently ate.

#### Barriers and facilitators towards consuming pulses following recipe provision

Eight themes were found following recipe provision: ‘Awareness’; ‘Willingness, trying new things’; ‘Small changes’; ‘Facilitators: Enjoyment, sensory properties, practical concerns, benefits’; ‘Facilitators: Knowledge, cooking ideas and confidence, incorporation, cooking solutions’; ‘Barriers: Risk and preconceptions’, ‘Barriers: Forgetting/laziness, habit, influence of others, compatibility, missing nutrients’, and ‘Barriers: Awareness, but’.

*Awareness*. All participants reported an increased awareness of pulses since completing earlier parts of each study. This increased awareness ranged from reminders of current or previous consumption, to increased conscious raising based on the range of benefits from pulse consumption, to also result in increased recognition that pulses were in the diet already, were available in habitual shopping venues, and to a more active consumption of pulses.

*Willingness, trying new things*. For some participants, increased awareness was also associated with increased willingness to consume more pulses, a willingness or openness to trying new things; a feeling accompanied by a sense of discovery or the suggestion of a welcome challenge. Participants also reported a willingness to try new things on observing the different dishes that could be made with pulses; recipes that were specifically selected to demonstrate variety and accommodate different tastes.

*Small changes*. Some participants reported ‘*trying to make changes’*, ‘*attempting to change’,* or making only small changes; feelings that were accompanied by less confidence and certainty than was associated with ‘willingness to change’. Dietary changes were typically small, such as trying one recipe or eating more of the pulses they already consumed, and many changes involved simply incorporating pulses into other dishes or meals, rather than creating new dishes. These dietary changes were largely considered positive, and the recipes were generally well received, but this wasn’t the case for everyone.

*Facilitators: enjoyment, sensory properties, practical concerns, benefits*. Barriers and facilitators to making changes were reported, as were given prior to receiving the recipes. Enjoyment, sensory properties, and practical concerns were again provided as barriers and facilitators, but there was a focus more on facilitative aspects than barriers. Participants mentioned the ease with which canned pulses could be used, the range of pulses that they could buy, and the ease with which pulses can be stored, which meant they were always available. Many benefits of pulse consumption were again reported, to include health benefits, nutritional benefits, benefits to meals by adding bulk, variety and satisfaction, and benefits to well-being as a result of self-care. Participants recognised multiple benefits from pulses, and some participants recognised that some benefits may be gained not so much from what was eaten, but from what was not eaten as a result. Participants did, however, also recognise that benefits may not be obvious or may be small, particularly if only small amounts of pulses were consumed.

*Facilitators: knowledge, cooking ideas and confidence, incorporation, cooking solutions*. Knowledge was reportedly increased, although further increases in knowledge were also desired. Increased practical knowledge particularly was mentioned; participants wanted more knowledge on what they could do with pulses, how to add them to their diet, and how to make them tasty. Participants mentioned a lack of cooking skills, a need for recipes and ideas, with again preferences for easy dishes or ones without too many ingredients. Participants recognised the ease with which pulses could be added to the diet through incorporation into existing dishes, and that cooking knowledge, ideas, and confidence could provide the solutions needed to address other barriers – that pulses could ‘*add benefit [to the diet] with no cost provided you can cook*’. Cooking solutions were provided to mask tastes or a lack of taste, and textures, but also to avoid the inconvenience of cooking with (dried) pulses, and other negative practical elements.

*Barriers: risk and preconceptions*. Some participants reported resistance to trying the recipes and new dishes, due to an element of risk. They were uninclined to make something that then may not taste nice so would be wasted, or may not be satisfying so would require supplementing. Many preconceptions or negative expectations of pulses were reported; suggestions that pulses would be ‘*bland’*, ‘*tasteless’*, would ‘*not add anything’*, and would be difficult, inconvenient or time consuming to use. These expectations were then largely countered by a positive experience and surprise when trying the recipes and tasting the dishes.

*Barriers: forgetting/laziness, habit, influence of others, compatibility, missing nutrients*. Other barriers to trying the recipes included forgetting or laziness, habit and an unwillingness to try anything new, or were attributed to the influence of others, due to food shopping responsibilities or likely consumption, although benefits of others were also given. Some participants again mentioned that pulses would be incompatible with other aspects of the diet, to include the possibility that nutrients may be missing from the diet if pulses replaced certain other items.

*Barriers: awareness, but ….* Some participants recognised that their awareness of pulses and their pulse consumption had increased, but that this hadn’t resulted in any changes in behaviour. Some participants mentioned that a further push, e.g. in the form of reminders, or requests from others, might encourage them, but also mentioned that they were currently unlikely to do anything on their own.

## Discussion

This work aimed to explore barriers and facilitators towards consuming pulses and towards increasing pulse consumption and any changes to these barriers and facilitators following requests to try some recipes at home.

Several barriers and facilitators towards pulse consumption were found. Many of these have been reported previously^(3,20–26)^, and many of these barriers and facilitators are also found in relation to the consumption of other healthy and sustainable foods^(35–49)^. Enjoyment, sensory properties, and health benefits are commonly reported as predictors of healthy sustainable food consumption^(36–42,44–49)^, and personal and external influences are also well recognised^(42,46,47)^. Of particular note in this study, are the themes related to practical concerns, cooking concerns, and compatibility with the current diet.

As facilitators of current consumption, the low cost, low wastage, and easy storage of pulses were recognised, and this has been suggested elsewhere^(25,26)^. Some participants also recognised the ease and convenience with which pulses could be used, but others suggested difficulties that pulses are inconvenient or time-consuming to prepare, that they lack sufficient knowledge to cook pulses, and that they lack the necessary skills and confidence. These cooking concerns were provided as barriers towards both current consumption and increasing consumption and are often reported in relation to pulses^(3,20–25)^. Similar concerns are also expressed specifically for vegetables^(38–40,42,45,47,49)^; another healthy food group that typically also requires preparing and cooking^(40,46)^. These concerns were reduced following recognition that canned pulses could be used and that pulses could be incorporated into existing dishes. The idea of incorporation was generally viewed positively, with participants recognising that the health, nutritional, and structural benefits of pulses could potentially be gained at little cost; cost, in this respect, not only in terms of finances and effort, but participants also recognised limited potential changes to taste or other desirable aspects of a dish. The ease with which pulses can be incorporated into dishes and the limited negative impacts of this incorporation on the dish as a whole have previously been mentioned when promoting pulse consumption for health benefits^(22,23)^.

Related to this, some participants also expressed concerns that it may not be appropriate to incorporate pulses into all dishes or into their existing diet, either now or in the future. Participants recognised the versatility of pulses, i.e. that they could be used in many different dishes, and this has again been suggested elsewhere^(20,26)^, but participants also suggested that pulses couldn’t necessarily substitute for other food items, such as animal products or vegetables. The idea that pulses may play a unique role in the dishes that include them, as a vegetable, a protein source or a carbohydrate source is interesting, but may present both an advantage and a challenge to increasing consumption^(5,21,25)^. In many countries, pulses are included in fruit and vegetable recommendations, but pulses are also recognised as a valuable protein source for those who wish to consume no or less meat, and as a source of complex carbohydrate^(21)^. The many nutritional benefits of pulses may potentially encourage consumption^(3,28)^, but perceptions of pulses as a ‘food for vegetarians’ have also previously been suggested to hinder consumption^(21)^.

Practical concerns and cooking concerns were considerably improved by trying some recipes. Practical aspects were mentioned almost entirely as facilitators following recipe provision, or were acknowledged, but without suggestion that they were barriers. Making the recipes was largely a positive experience, sometimes with some considerable surprise. Participants varied in the recipes they tried, from not trying any to trying several, both those provided and others, and the recipes ranged from simple adaptions to existing dishes to using pulses in sweet foods such as cakes and pulse-centred dishes from alternative cuisines. Some participants welcomed comprehensive ideas, to discover new dishes and challenge themselves, determined to make positive changes, while others reported a preference and desire for easy solutions, and showed a preference for small changes, accompanied by a lack of certainty and confidence. A preference for small changes to increase sustainable eating has previously been suggested^(26,36,37,46)^ and the idea that pulses could be added to the diet for multiple benefits with no or very little cost was appealing. Increased practical knowledge, more recipe ideas, cooking skills and confidence were requested by many and suggested to be valuable. Many participants also recognised these as practical solutions for many of their previous barriers, particularly those related to sensory properties. The value of cooking knowledge, skills, and recipes for improving the sensory properties of healthy foods is well known^(3,28,50)^. The value of the recipes for improving perceptions of pulse consumption is a unique insight gained from this research.

While participants appreciated greater practical knowledge, many participants also referred to benefits or asked for more academic knowledge, suggesting that recipes and practical advice alone may not be enough, that some reason for trying and cooking recipes was also required. Coupled with this knowledge of benefits, some participants also suggested value from an increased awareness of pulses, an increased awareness of their own consumption and value from increased awareness of recommendations. These comments have again been reported previously, both in relation to pulse consumption^(20,21,25)^ and in relation to healthy and sustainable eating^(37,41,45–48)^, but academic or theoretical knowledge is often suggested as insufficient on its own to result in behaviour change^(20,21,25,26,46,48)^. Our data suggest a clear complementarity to both academic and practical knowledge and awareness.

In relation to both knowledge and awareness, it was also noticeable that many participants held preconceptions or expectations that pulse consumption would be a negative experience. Participants were surprised at the pleasure gained from the dishes they had made that included pulses, and suspicions or expectations of a negative experience were given as reasons for not trying recipes. For some participants, low expectations coupled with the effort and inconvenience of pulses resulted in too great a risk for them to try the recipes. Low expectations of pulses as dull, tasteless, and gassy have been reported^(3,20,23)^, although in our sample few concerns over gastric discomfort or distress were offered. Flatulence and intestinal discomfort are more commonly reported elsewhere^(20,24)^, but there are suggestions that modern canning and preparatory activities can reduce these effects^(3,23,24,26)^. The intestinal effects of pulse consumption are also thought to decrease with experience^(20,24,26)^, and notably, in systematic reviews of health impacts, few adverse events of pulse consumption are reported^(2)^. The practices associated with reduced intestinal discomfort may also increase the health and nutritional value of pulses^(3)^. Some bioactive compounds present in pulses such as lectins and protease inhibitors can interfere with the digestion and utilisation of other nutrients^(3,4)^, and preparation and cooking activities such as soaking, boiling, and steaming are known to reduce this interference^(3,4)^.

Despite these preconceptions or expectations, participants in our sample were generally willing to try the recipes or consume more pulses, as has also been found elsewhere^(22,26)^. This is possibly a reflection of study participation^(30)^, but this willingness also did not apply to everyone. Poor compatibility with existing diets remained a concern for some participants, while others simply forgot, mentioned competing demands, or could offer no explanation. While pulses may seem an ideal food from many perspectives, there will always be a need to respect individual differences and preferences.

A number of suggestions for encouraging pulse consumption can be offered from our study. First, education on the multiple benefits of pulses will increase awareness, provide information, and provide reasons for increased consumption^(3,21,24,26)^. A recommended amount to consume and acknowledgement of the benefits gained from small portions may also be of value^(24)^. Health benefits are reported in the literature from portions of 63–81 g/d^(10)^, 120–132g/d,^(9)^ and 150g/d^(2)^, roughly ¼ to ½ a standard 400g can when drained. Some education may also be needed on the place of pulses in the diet, i.e. as a vegetable, protein source, or carbohydrate source^(21)^. Second, some practical suggestions may encourage action. A range of easy cooking tips, such as incorporating pulses into soups and stews, will facilitate small changes and appeal to those with low confidence, commitment, or willingness to try^(22,24–26,28)^. A range of pulse-based recipes from cultures that traditionally use pulses will challenge those with more confidence to discover new experiences^(21,24,26)^. Others have also suggested that highlighting the many and varied dishes that can include pulses may be of value, particularly for those who are less familiar with them^(20,26)^. Third, some activities to challenge preconceptions and increase familiarity may also be of value. These activities could range from increasing awareness of the pulses already contained in popular dishes, such as the beans in chillis or the chickpeas in houmous^(20,22,24,43)^, to adding pulses to traditional meat-based recipes on a gradual basis to increase acceptability^(23,28)^, free food tastings and tasty food offerings^(22,43)^, and encouraging the provision of pulse-based dishes in scenarios where preparation and cooking are not required, i.e. in out-of-home food outlets such as workplace canteens and restaurants^(21,22,24,43)^.

Strengths of our research are the inclusion of a range of participants with a range of experiences with pulses, and the use of in-depth interviews to provide a range of opinions. Our research is limited by the necessary small and select sample^(30)^, limiting the analyses and conclusions that can be made^(30,34)^. Most notably, our studies were set up to include a request to try some recipes, thus there may have been some volunteer bias, such that we were more likely to have included volunteers who enjoyed cooking or thought of this positively. Not all participants, however, complied with the request to try recipes, thus the impact of this bias may only be small. A population-wide survey allowing capture of opinions from a much wider sample would be of value. Our research was also conducted in the UK, during winter time. Pulses are not currently commonly consumed in the UK, with the exception of baked beans, and this lack of familiarity may have increased the negative perceptions of pulses. Previous studies, however, suggest that pulses are consumed more in winter or may be more acceptable at this time of year^(20)^, thus season may have impacted positively on our findings. Our studies were also conducted during the COVID-19 pandemic when some lifestyle restrictions were in place, and an increase in home cooking and home working during this period may have resulted in an increased awareness of these concerns and a greater willingness to consider increased cooking activities. We cannot assess the impact of this potential bias in this dataset, but further study on a wider scale would also reduce these concerns.

In conclusion, this qualitative research finds a number of barriers and facilitators towards pulse consumption, including themes relating to ‘practical concerns’, ‘cooking concerns’, and ‘compatibility with current diet’. Similar barriers and facilitators to increasing pulse consumption were also found. Provision of some simple cooking suggestions and pulse-based recipes resulted in themes on ‘Awareness’; ‘Willingness, Trying New Things’; ‘Small Changes’ and facilitators associated with ‘Enjoyment, Sensory Properties, Practical Concerns, Benefits’ and ‘Knowledge, Cooking Ideas and Confidence, Incorporation, Cooking Solutions’. Barriers related to ‘Risk and Preconceptions’; ‘Awareness, but’ inaction and additional considerations were also found. Our findings demonstrate a positive role in pulse consumption for increased experience, familiarity, and confidence with preparing, cooking, and consuming these healthy, sustainable foods. Moving forward, we suggest a need for increased education on the many benefits of pulses to increase awareness and provide reasons for consumption; provision of practical suggestions, from incorporation advice for those less confident to pulse-based recipes for those looking for new experiences; and activities or opportunities to increase familiarity and reduce preconceptions.

## Supplementary material

For supplementary material accompanying this paper visit https://doi.org/10.1017/jns.2023.119. click here to view supplementary material
