# Supplementary material for: Barriers and facilitators to consuming pulses: a qualitative exploration including effects of trying recipes at home
Source: J Nutr Sci. 2024 Jan 31;13:e6. doi: 10.1017/jns.2023.119 (PMC10877139; doi:10.1017/jns.2023.119)
Supplement: Supplementary file 1 [file S2048679023001192sup001.docx]

**BARRIERS AND FACILITATORS TO CONSUMING PULSES: A QUALITATIVE EXPLORATION INCLUDING EFFECTS OF TRYING RECIPES AT HOME**

**Whittall B, Warwick SM, Jackson M, Appleton KM.**

**Research Centre for Behaviour Change, Department of Psychology, Bournemouth University, UK**

**Codes and quotes per theme and sub-theme for each of the three research aims, as gained from interviews from both studies.**

**3.2.1. Supplementary: BARRIERS AND FACILITATORS TOWARD CONSUMING PULSES**

**Enjoyment**

Liking - I actually really like lentils, so I thought I would try it, and yeah, it doesn’t taste too bad (B14)

Liking - I'm not a massive fan of pulses and stuff, but in your example you said beans and there's quite a range of beans, but I like quite a lot of beans, but I'm not a massive fan of lentils and stuff like that (B19)

Liking - we don't really think about the health benefits, we just eat them because we like them (B21)

**Sensory Properties**

Taste - they have a good taste (B1)

Taste - they just don't taste very good (B19)

Taste - I think it’s important for me to find the one that I like the taste of (B18)

Taste - as long as it's tasty I'll have it (B15)

Flavour - I don’t think they add anything in terms of flavour (B8)

Texture - and it’s just nice to have a different texture (B10)

Texture - other than kidney beans, I just find them a bit dry but I do still eat them (B17)

Texture - There are some beans that I really don’t like so I don’t eat baked beans, but especially with kidney beans I don’t really like the texture of them, so for me if I was making a chilli con carney I just wouldn’t put them in (B8)

Taste - Again, it comes down to my cooking or lack of, but they are a bit tasteless unless you put them in something that’s nice. So, you have to put a sauce or gravy or something with them. Or include them in something that is quite tasty. But a bowl of lentil soup doesn’t incline me really. And I’m not inclined to put in a lot of salt or pepper to make it tasty when it should already be tasty. Or I have an option, that I think is more tasty. So I look at lentil soup and think, nah, I’ll pass on that. I’ll have the tomato and basil (B11)

**Benefits**

Nutritional benefits - So yeah, it would be an extra way to get more protein in, and I think protein is incredible (B1)

Nutritional benefits - they’re good for fibre and protein, and I feel like they would make you fill up for longer (B3)

Nutritional composition - And then I think the other thing would be the amount of protein in them. I know that they say that it’s high in protein but I do feel like they do have a lot of carbs in them as well, where it is easier to have a piece of meat or an egg which doesn’t have carbohydrates in. Whereas when you have lentils or pulses in your meal it does add protein to it but it does add a lot of carbohydrates to it as well, and I struggle with that (B7)

Health/nutritional benefits - A good wholesome food and high in protein, so generally just really healthy (B4)

Nutritional benefits - it actually filled me up but also not adding extra carbohydrate to it, and you can also get protein and fibre from it (B12)

Nutritional benefits - would obviously be the natural fibre that is in there, the other vitamins, etc., that are included (B11)

Nutritional benefits - there's a lot of belief that you don't really need to eat meat and you can still get the same proteins and what not from plants and pulses I guess (B19)

Dietary component - I found it was a really good way to get my veg, because sometimes I was tired I was eating a lot of chicken and rice but not loads of veg, but with chillis, I felt like I could just chuck in a lot of different veg and it tasted really nice (B6)

Well-being - I was thinking I could make something like houmous as a snack I think that would be a nice feeling and healthy and relatively cheap snack to have, making houmous (B14)

Flatulence - can make you gassy sometimes (B16)

Fillingness - probably incorporating them into recipes makes the meal more substantial in terms of filling you up and a bit of a heavier a meal. So, it can fill you up for longer but they can also provide health benefits as well (B12)

Adds bulk – bulk out food (B4)

Adds bulk - then I’d put them in food that I’m already eating to help boost that up. I do that quite regularly with a green peas but there are a lot more food especially in London like lentils which I could incorporate into my foods. So, these are possible and they could bulk out the meal (B18)

Natural - I suppose having anything natural really isn’t anything negative (B8)

Safety - I think there are some that you have to wash particularly carefully because of some thing but obviously I’ve never paid much attention to it so I haven’t worried about it. But maybe some people are a bit wary about eating them (B10)

**Recommendations**

Portion size knowledge - the amount you’re supposed to have a week (B7)

Portion sizes - I think that’s quite beneficial that you don’t need to have that much to get the benefits. Obviously, I do presume it’s the case of the more the merrier. But you could always have a lot less if you wanted to or you can always eat a lot more and it wouldn’t do any harm (B8)

Portion sizes - I didn’t realise, I would of thought, if you needed them for a healthy diet you would need them everyday, but the lady in the video recommends only three times a week to get the benefits. I would presume you needed more of them (B15)

Recommendations - I didn’t know you were supposed to eat it each week (B16)

**Practical concerns**

Availability - I guess availability in small shops, if you just popped up to the convenience store I don’t know if they sell them. But most convenient stores if you have forgot to pick up some chicken they probably have in there (B14)

Availability / Variety - sometimes I use a variety of beans in chillis (B6)

Cost - they are definitely much cheaper (B14)

Cost - they are an inexpensive protein, so it would make a great alternative to meat and it is more accessible to people who can’t afford the alternatives, that are too expensive (B6)

Cost - it sounds like they are quite cheap (B4)

Cost - personally I think they are quite cheap to buy (B7)

Storage - they go along way - you can get a big bag and you only need a cup or two for a recipe, and then you have loads left, so that’s a huge benefit (B14)

Storage - You can store them for a long time also which may help, with seasonal things. Like way back when, people were living off, well, they are a sustainable food, I guess. You can have them dried, so you have it fresh and then you dry it, and that’s probably what my grandmother, would probably do to that, more than my mother. For sure, because of the need. No fridges or freezers that sort of thing (B11)

Storage - you can get them in tins so they last longer as well, and we have them in the cupboard as well, so they're not really going to be perishable necessarily. But other foods like meat and stuff are a lot more perishable (B13)

**Cooking concerns: Easy, Convenient, Versatile**

Easy - I think that they are quite easy to use, but in different recipes (B7)

Easy - when I am going to a supermarket, they do these prepacked pulses where you can just get a Mediterranean grain or a mid eastern grain mix and they’re really nice to add into a salad (B10)

Easy - Garden peas are something that I have all the time, as it is the most easiest vegetable to prepare. That is something I have probably maybe even twice a day, just because they are so easy just to stick in a saucepan and boil up and it’s just done (B18)

Easy - they are just so easy (B10)

Convenience - Because I buy them tinned or in those precooked packets there is an element of convenience that comes with them, although I was cooking something and I had a type of lentil and I was adding it into a sauce and I was really surprised how quickly it cooked, and I realise that I probably should stop being so lazy and save some money and buy dried because it is really affordable (B10)

Effort - I guess its just laziness, and routine, and how I was brought up I guess. What you get used to eating (B11)

Versatile - there’s so many different things you can do with it to make it really delicious (B10)

Versatile - I think they are quite versatile and there are quite a lot of different ways you can use it (B7)

Versatility - there is a different variety of pulses that you can have so you can have one that you quite like (B4)

Versatility - Obviously it has good cooking potential. You can put them in lots of different things (B1)

Versatile - I think because we have them in different ways (B21)

Versatile - I was thinking I could make something like houmous as a snack (B14)

Versatile - if I have a long day at work then I might just go into the fridge and eat a spoonful of chickpeas (B1)

**Cooking concerns: Planning and Preparation**

Preparation time - And then I will say for the negative points for me, it would be where you buy them dry and they can be quite hard to soak, so more prep time to be able to cook them properly (B7)

Planning - I guess with some beans if you have to buy them dry and soak them in water but then I guess people would just buy the ones in the tin and you can just use straight away, but that would be about it for me it’s going to take four hours to soak to them, I’m not planning my meal that far in advance (B14)

Effort / planning - I guess it’s just because I’m a bit lazy and I don’t always want to soak them, when you buy the dry ones you have to save them for such a matter of time before you cook them so I guess as a laziness factor for that, but I just buy the tinned ones instead so that kind of cancels that out which is pretty good (B10)

Planning - So I don’t plan my meals that much so if I was going to try and incorporate them I would probably have to plan my meals a bit more (B8)

**Cooking concerns: Cooking Knowledge**

Preparation knowledge - I feel like there’s not a lot of well-known recipes out there. I mean I’m sure there are, but I don’t know enough about good recipes for pulses. So I don’t feel like there’s anything obvious that I would use to make, other than chilies which I do really enjoy, I’d need to know some more decent recipes (B6)

Preparation knowledge - I just think that there is not enough information on how to include them. We are so used to the easiest options available to us. And the ones that we know. And I don’t think there was much information whilst at school on pulses and how to add them to dishes. I’m so used to looking online and seeing how to fry a bit of chicken, or chucking meat in the oven or boiling potatoes. But these pulses require a lot of different ways to cook and they’re not really taught. And I think they are different levels of cooking, it’s not the same as normal. So I think there needs to be more information on how to use them so that we can book them out in meals we already eat (B18)

Preparation knowledge - I need to know how to make it, and how much to put in of each ingredient and when (B18)

**Cooking concerns: Incorporation**

Incorporated – If it’s part of a meal, then yeah, I’ll eat it (B1)

Incorporated - I would not eat them on their own (B1)

Incorporated - So yeah, I actually eat them everyday, well pretty much every day, they are part of my stew type thing that I have for lunch, I also have all sorts of beans and peas and what not in there. We buy them in tins so tinned chickpeas or black beans (B1)

Hiding - they're quite easy to hide in a meal as well. So, if you're trying to get someone to eat more you can put them in without them knowing (B1)

Incorporation - We don’t really cook things from fresh so it could be quite hard to add them to a meal at the moment (B3)

Incorporation - You can put them in different types of food as well, which sounds quite good (B4)

Incorporation - I think I would probably eat them, if they weren’t just whole and if they were blended in, so I can see them being used a lot more in that respect (B8)

Incorporation - I would have to incorporate them in a way that the lady did with the soup where they are sort of blended and put in that way, because for me I just wouldn’t want to eat beans how they are because like I say I’m not a massive fan of them. But if they’re all mixed up within something that wouldn’t bother me and I would be fine with it (B8)

Incorporation - Like I said we put them in different types of curries, and see if we have things like lentil soup. Different beans we kind of make into …, like, red kidney beans that we put into separate curries, or we will put them in our Mexican meals and have them in enchiladas. I think we tried to have them consistently throughout the week if possible (B21)

Incorporation - I will eat them with peas and that, and when I went to Thailand there was a lot of foods with pulses and beans in the food, and you just sort of eat it as it was part of the meal (B19)

Incorporation - we sometimes have them in soups (B20)

Incorporation - I don't think I'm overly keen about having them just by themselves, but in things like curries they are absolutely fine, and I've eaten a few just by themselves, but they're not necessarily as good or tasty, but in curries and dinners and different sauces, they're fine (B13)

Incorporation - they seem like they are really good to be able to just put into your food and just adding to different foods you already eat (B20)

**Cooking concerns: Alternatives / Replacements / Substitutes**

Alternative - Like I said I am a pretty big meat eater, but I do you think we could all reduce our meat, and with mince it’s more like a texture thing rather than having a steak which would be pretty hard to find a meatless replacement (B14)

Alternatives - with things like the Bolognese and the chilli I think you can substitute it without it making a massive impact on the meal (B14)

Alternatives - I like the taste of meat and I couldn’t be just vegetarian or go with a pulse diet because they’re not going to substitute a chicken breast. So, for some meals you can replace it but some they can’t (B14)

Replacement - I tend to not to, rather than use, say lentils or anything in a casserole, I will put in more vegetables (B11)

Alternative/Replacement - they are worth while eating because meat is all well and good but you can only eat so much meat in the day and you start sweating and getting meat sweats or whatever, and so it’s not always brilliant for you (B1)

Replacement - I think after watching that they are quite beneficial especially if people stop them in for something, I think they could be quite beneficial (B7)

Substitute - I kind of get it now especially if they can be used as a substitute. I’m not sure I will put them in a Brownie, I think that’s a little bit far but I get that when she’s having a soup instead of putting milk put some white beans in there, it did sort of makes sense in that respect (B8)

Replacement - I wouldn't have them too much because they wouldn't replace vegetables (B21)

**Compatibility with current diet**

Compatible with existing diet - I don’t mind experimenting and having a chickpea curry or something but I would like to have a chicken korma as well and have a bit of both (B14)

Compatibility with current diet - I don’t know how they would fit into something that I usually make (B9)

Compatibility with current diet - I would probably have to change my meals a little bit because there’s not a lot of meals which I would have that in (B4)

Compatibility with current diet - For me I think the only bad thing really would be a change in the way I cook, and change in my diet, our diets (B11)

Compatible with existing diet - those brownies I’ll give them a go, but I do love a normal brownie (B14)

Giving up existing foods - I like the taste of meat is my main barrier for not wanting to have them every day (B14)

Tempting alternatives - Temptations around me at the moment, almost like the ‘what for?’ attitude everyone has at the moment, but that's just because of lockdown (B19)

**Dietary choices / restrictions**

Dietary choices / restrictions - I definitely think I use them a lot more now than I did before I was gluten-free, it was something I made a very conscious swap on and I eat things more like quinoa and chickpeas and beans, like if I was doing a veggie chilli I’ll just do a bean chilli type of thing, and I found that that is something that actually fills me up and it’s not just a vegetable (B12)

Dietary choices / restrictions - Being vegetarian at one point means that a lot of them were in my diet from vegetarian cooking, so I was introduced to them there. And even though I am not veggie anymore I still use them in my dishes (B16)

**Personal Influences: Familiarity and Habits**

Habit - I would say that I cook more with them (B3)

Familiarity - I guess they’re really common in a lot of international dishes so where we’re trying to be experimental with meals, I will add them into a lot of things (B10)

Familiar - there’s quite a lot of things like beans that go into like a chilli con carney so like kidney beans (B8)

Familiar - I have used them before, yeah. One of my favourite things I’d like to make is a lentil bolognese, that can be really nice sometimes (B7)

Familiar - I wouldn’t say it’s something that I would use that regularly, but it is something I do (B7)

Habit - I used to eat a pretty similar diet, I used to eat a lot of chillis, so I used to have different types of beans in the chillis (B6)

Habit - I guess I use them when I make chilli, it’s quite often (B6)

Staple - also going back to travelling and eating food from lots of different countries, it’s a staple in a lot of different countries and a lot of different recipes … particular being in Mexico and Central America if you had a meal without some beans you think what’s going on (B10)

Familiar - surprisingly we actually eat lentils a fair bit, probably twice a week I’d say we have lentils. We have this sweet potato curry, sweet potato and lentil curry, we also have a lentil Bolognese, which is taking out the meat and replacing it with lentils which tastes identical. Then, beans I probably don’t eat that many beans but I do use them mainly in chillis, so that’s when we mainly eat beans, but your lentils probably twice a week (B14)

Familiarity – we eat quite a lot of well a vegetarian diet really, so we use lentils quite a lot and we do bean chillis (B17)

Staple ­- it is a household food, everyone has a tin of baked beans in their cupboard (B2)

Experience - I don’t really have much experiences with pulses (B5)

Habit - I think they already are incorporated into my diet, when they were saying to get three cups for the week, I realise that I had got that without even thinking or realising I was doing it with just the meals I was cooking (B10)

**Personal Influences: Prior Knowledge**

Limited knowledge - I don’t really know what they are too much (B5)

Knowledge - I guess that was like me that when you asked me I was a bit unsure, so I guess there is a lot of people that probably don’t know what pulses are (B10)

Knowledge - I didn't really know much about pulses (B21)

Knowledge - I was a vegetarian so I knew they were a good protein substitute (B16)

Already do, but unaware - I guess, we kind of do I guess we eat quite a lot of them, especially if we are making a veggie curry we always put kidney beans in. In fajitas, we always put black beans in, and so we do eat them quite a lot (B13)

Knowledge - But you know, that’s only really from ignorance over anything else (B11)

Knowledge - I did know a lot of that already, I don’t think there’s anything I didn’t know (B17)

**Personal Influences: Upbringing and childhood experiences**

Previous experiences - I didn’t really try things when I was younger (B3)

Upbringing - I think it’s just how I brought up. We never used to have them because – we didn’t (B11)

Upbringing - I think I’ve just grown up with them, I really liked them (B10)

Upbringing - But as my grandma would feed me meals with pulses I’ve kind of grown up loving them so it’s never been an issue for me (B10)

**Personal Influences: Personal preferences**

Individual differences - they are not for everyone (B16)

Adventurous - lentils and chickpeas are used in curries. Beans in Mexican cooking like chillis, but I haven't really ventured out from that (B16)

**External influences**

Parental influences - it’s from seeing what your parents eat. I think that’s what I’ve been figuring out recently as my mum doesn’t really try anything new (B3)

Influence of others - when I met my partner he has all this amazing food and I’m like, all that looks good and then I try it and I say why haven’t I had this more (B3)

Influence of others - But his parents cook more with them when we live with them (B3)

Influence of others - my sister actually. She is wanting to do one meat free day a week, So she’s trying out all these recipes and when they are good she is sending them along (B14)

Influence of others - Someone I used to live with used to cook a lot of different pulses. They used to be really into their pulses. His partner is a vegetarian so he doesn’t really eat much meat. It’s not that he doesn’t want to eat meat, but it’s just easier to make vegetarian food, and he used to make some really nice dishes with pulses, really nice food actually. So, I guess I have a really good experiences of pulses (B6)

Influence of others - he used to make things like lentil curry is lots of lentil curries, he would do things with black bean curries and he used to make a lot of falafel, which was really delicious. We used to have falafel wraps and that was really, really nice, so I have had a few different types of pulses (B6)

Influence of others - we’re not really a pulse family (B5)

Influence of others - I think it was my grandma’s influence as she used to eat a lot of pulses (B10)

External influences - the other recipe my partner found and I think we saw it on a menu at a restaurant and we thought that we would like to try that, so found a recipe online (B14)

External influences - I think I must of seen them in a video or something (B20)

External influences - A few people that I follow on Instagram, who are quite into their health and the athletics, they are starting to use a lot more pulses, and pulse based diets, and replacing a lot of things with pulses. It's a lot more plant-based these days isn't it? (B19)

Influence of others - I guess if it’s …, if my partner didn’t want to eat them then that would be a barrier, because we are both eating the same meals. And I’m only living with one person but if you were living with the family and if there was someone that was really against eating them - I’m really fussy, that would definitely be a barrier, but luckily he’s pretty willing to try things (B14)

Cooking for others - But when we have people round for food, you generally you stick to meals you know people are going to be like, so I guess I could be a barrier (B14)

**3.2.2. Supplementary: BARRIERS AND FACILITATORS TOWARDS CONSUMING MORE PULSES**

**Willingness**

Willingness - I would potentially attempt to cook something (B5)

Willingness - I don’t know what they taste like, but I would be willing to try it (B5)

Willingness - I would be up for ordering some and giving it a go (B11)

Willingness - I think I already used them before and I think I will continue to use them (B12)

Previous experience - I used to eat beans quite a lot anyway (B2)

Willingness - I am interested in using them more and in different ways (B12)

Willingness - I don't suppose you have the recipe for the Cincinnati chilli do you? I really fancy giving that a go (B15)

Willingness - I'd like to, I'd really like to. I really want to try that Brownie, I don't think it's going to be nice, but I still want to try it to see if it is (B20)

Willingness - I might try some different ones (B17)

Willingness - I would give it a go, surprisingly your call has prompted me to investigate eating healthier, so yeah (B5)

Adventurous - I also need to try an experiment a bit and push the boat out a bit so this could be a good way (B19)

**Knowledge of Benefits**

Nutritional composition - I would say knowing exactly what to pair them with to make a balanced meal. Say one of the recipes for example on the video was a Cincinnati Chilli with the spaghetti, it would probably be better to not have the spaghetti with it, as there is quite a lot of carbohydrates in that. So, it will be knowing the exact breakdown (B7)

Health benefits - having the knowledge that it is healthy for you would probably encourage me to eat it a little bit more (B2)

Knowledge - I think the education behind diet and food isn’t enough to help people make the right decisions (B2)

Nutritional benefits - it looks like they are lower calories (B5)

Filling - I feel like they would fill you up with less calories compared to another product (B5)

Nutritional benefits - I’d say they be really beneficial for my diet because I need a slightly more varied diet. At the moment I feel like I’m eating a lot of the same things (B6)

Nutritional knowledge - the nutritional value and the breakdown of them, and all the different types because there is quite a lot and finding out which ones are more balanced with their macronutrients (B7)

Health benefits - it’s probably going to go towards making me feel less lethargic, or just giving me a little bit more energy, I’m all for lasting energy (B8)

Benefits - to add some bulk, and something different (B20)

Benefits - I just eat what I like and as a family, but now I know I can actually think about how to use them as they are quite beneficial, and I would be a bit more mindful about it (B21)

Benefits - I think I’d definitely eat them, more likely now I know the positive things about them. I think I'd make more of a conscious effort to buy them a bit more and stuff. I guess it's nice because now I know that when I'm cooking with them they are more sustainable and there's a lot more protein in them, so that's good to know (B12)

Knowledge - I suppose it’s probably knowledge of what pulses, I know more now. You are always more comfortable with things you know (B15)

Benefits - as the years go on I want to work less and I want to get more into my diet, and be more specific with what I'm doing and that (B20)

Health benefits - I think I probably would do, I think it would be interesting to see any other benefits that I didn't know about like the cancer, like reducing breast cancer as I didn't really know that. might be other things that they are really beneficial for (B13)

Benefits - If I was going to make a dish and see what the health benefits was going to be I would look at the recipes as a whole, or maybe look at individual elements, I wouldn’t specifically go into pulses. But I would be more inclined to do it with something like pulses I guess because I know it’s something that I am less likely to like so I would want to find more benefits for doing it. So if I didn’t know about any benefits of it I wouldn’t bother putting it in there. Whereas if I could find a load of benefits on it I might be like, oh yeah, okay, I could put them in there and then, and hopefully. I will like it more because it’s going to benefit me so much more for putting them in it (B8)

Benefits - potentially, if I’m looking at getting fit and healthy and trying to control my diet a little bit more them for sure I would probably do my own research, but I just have to be in that mindset of wanting to (B2)

Nutritional composition - I think that I do have a lot of friends that are vegetarian or vegan, so when they’re coming round for dinner in a non-Covid world I do try to think about them having a balanced meal and having enough, I’m just making sure they get the protein element in there (B10)

Environmental concerns - I would like to do some more research, especially if they are more sustainable with the nitrogen and the fertiliser, I think I would do some more research into how sustainable are they, and should we be more conscious about where with it we are buying them from (B12)

More knowledge - I guess I would do a little bit of research probably (B3)

More knowledge – then, I would probably research more about them (B9)

Knowledge for others - it would be good to get a confirmation of what my mum makes is very healthy, and it would be good for her to know as well. I think it's like a lot of things get passed down, so I think it's a cultural thing that people might of used to know years ago, but now we just kind of have them (B21)

**Awareness**

Awareness - I could eat a few more different things in my diet. But to be honest I’ll probably make a chilli because I haven’t done in ages and I’ve been thinking about it now (B6)

Reminder - now I do remember them I would buy them because they are cheaper, and they are sustainable source and things (B7)

**Sufficient Academic Knowledge**

Sufficient knowledge - Would I do any more research? Probably not. Deep down I probably know I should eat more fibre, and they do naturally bring fibre into your diet, so you know (B11)

Sufficient knowledge - I wouldn't read any more into it because I already know that they are good and I used them and eat them (B1)

Sufficient knowledge - I don’t think I would look much more into the benefits (B4)

**Knowledge of Cooking**

Cooking knowledge - I know we have lots of recipe books that don't include pulses. So I would have to go looking for them, instead of them being naturally there in our house (B15)

Cooking knowledge - So we would have to find some more cookbooks. I don’t think pulses tend to leap out at you. And you might still have the belief that they are a bit boring, not that they are, but it could be argued that they are just seeds that need flavouring added to them (B15)

Cooking knowledge - I think also cooking it would be the main barrier because I don’t really know what to do with them (B5)

Preparation knowledge - If I saw a really good recipe somewhere or a video of something which looks really tasty that would motivate me to go and buy some beans (B6)

Preparation knowledge - I think it is just the making them taste nice in food, it’s not that they taste appalling but I like my food to taste really good, I don’t just eat things for the sake of it because I’m hungry I like a lot of flavour in my food (B6)

Cooking knowledge - Then I would also like to look at the best ways to use them to get the most benefits out of them. So that can be an issue to, so they might have those benefits that you might have to cook them in a certain way (B7)

Replacements - you can use them for alternatives to certain meat-based things (B5)

Cooking knowledge - But also, I would have to have an easy way of making them tasty. A convenient way of making them tasty (B11)

Practical knowledge - I could say for example chickpeas and think what chickpeas go with in a dish for example. Then I might put in some more research to find out what to do with it and how to buy it (B18)

Practical knowledge - I don't think I would research any more of the health benefits, but if we are going to include the more in our food, I would definitely look into more what you could do with it. (B20)

Cooking ideas - I would probably look into more about what you can cook with them (B4)

Cooking ideas - I would probably just look for more ideas on how to incorporate more into my diet (B4)

Cooking ideas - I think I would be tempted to look into some bean recipes, and I really like lentil soup so I probably look up a video on how to make a nice lentil soup and try something new? (B6)

Confidence - I suppose I will be a bit more confident about going out and seeking them out and trying them (B15)

**Practical Concerns**

Tasty – I would say that vegetarian food is more tasty than traditional food. So yeah, definitely interesting, would I explore more from that video? Yeah, I probably would. I thought the chilli looked delicious, and I think I will give that a go to see how tasty it is compared to a beef chilli (B15)

Easy - it looks quite easy to make and it looks like quite a simple replacement really (B2)

Easy - If it’s part of my diet it’s not hard to go out and find it (B2)

Easy - I can just add it to my diet and get healthier and be better for my body (B2)

Cost - I was saying about cost, if it is a replacement for meat it’s probably going to be more cost-effective, but if it’s something to add to or an addition to meal then it’s an extra cost really (B2)

Easy - I would put them in a few different types of food really, so yeah, I would incorporate more into my diet (B4)

Time - if I had the time to do it (B8)

Ease / effort - If I’m already eating something that’s premade then it would be pretty hard but if I was going out to buy dishes then I could get things that have them already incorporated in them or if I was going through the hassle of making something anyway then I wouldn’t have an issue of putting some in if I’m going through that effort anyway, it’s not any more effort to put any more in it, So I guess I could but I probably wouldn’t go massively out of my way to do it but I would definitely do it if I was already going out my way anyway (B8)

Effort / ease - if I was going to do it I would say at least once I’m sure I could do it, because I will probably make myself something from scratch at least once, but yeah once or twice I imagine. Maybe more but that’s probably unlikely but like I say that’s probably because I don’t make a lot of dishes from scratch (B8)

Ease / taste - if I give it ago, and I can make it easily into my diet, and the flavour isn’t, you know, changed too much (B11)

Easy - I think it would be so easy to put kidney beans and chickpeas and lentils to put into a casserole, to add some Bulk, and something different. It would be really easy to add it to foods that we already eat a lot of (B20)

Effort - making that actual change in how I shop, and how I cook (B11)

Addition - they’re not really taking anything else away, they are just adding it to current foods (B2)

Availability – I’ll have to buy some. I can’t honestly say I have any sitting in my cupboard, so I will need to get some and then try and incorporate it (B11)

Preferences - I’m just really fussy. So, then I will just stick to what I know because I know what I like (B5)

Taste – [if] they tasted really bad (B5)

Taste - the taste, if I try it and I don’t like the taste of it and it’s quite a weird taste, then it will make me take it out and I will have bad feelings towards doing that (B18)

Accessibility - I don’t have a car at the moment so there’s not even a shop where I am (B2)

Influence of others - I do need to ask my chef (B17)

Social influences - I’m living with my family food wise I do eat quite a lot what’s given to me (B2)

Influence of others / living circumstances - probably more so when I go to Uni, because then I’ll be in more control of my own diet (B5)

Influence of others - my mum doesn’t like beans, so any meal that she makes they are not going to be in (B8)

Influence of others - I'd like to, but I need to check with my parents what is on the shopping list (B20)

Influence of others - I wouldn’t be able to make it for my mum because she wouldn’t want it, so it would be just working out how to make a single portion of something, or making a big portion and then just freezing it (B8)

Living circumstances – the free range to do it as it is a small kitchen and you don’t have room for two of you to make dishes in there at once (B8)

**Compatibility with current diet**

Room - I just don't know if I could (B1)

Suitability - I'm not going to eat them for every meal, so like for breakfast (B1)

Incompatibility - If they didn’t go with what you are going to eat (B3)

Room - my mum‘s partner chucks loads of pulses in for my lunches so I probably do have them daily, so I probably couldn’t incorporate any more than that (B4)

Room - It’s probably in at least two of my meals a day (B4)

Sufficient already - I think it's probably we already have them, so we probably won't feel the need to have anymore (B21)

Suitability - if I eat them and I like them, and they can adapt to what I eat, so if I put them in something I like them, yeah, I would probably get some more (B9)

Compatible with current diet - you don’t really know how it’s going to complement the dish (B18)

Suitability - I think in terms of using them for sweet or in brownies I will definitely have to try something like that, but I definitely would put them in savoury meals and stuff like that, but I don’t know how much I would use them in sweet things. I’ll have to try and see (B11)

Suitability - If somebody had mentioned pulses to me, I may of thought they were someone who was completely a vegetarian or something. I wouldn't of twigged that you could incorporate it into all diet (B15)

**3.2.3. Supplementary: BARRIERS AND FACILITATORS TOWARDS CONSUMING PULSES FOLLOWING COOKING SUGGESTIONS AND RECIPE PROVISION**

**Awareness**

Awareness with interview - I would say that I have wanted to and I have definitely thought about it a lot more, I think even just talking about it … but even just talking about it makes you consider maybe you do need to eat more healthily to just feel better (B2)

Awareness - although I didn’t know about the name before I do now know about them, and I hadn’t put very much thought into it but since last time I have thought about it a bit more (B2)

Awareness - I would definitely say [my eating habits] have changed. Learning more about it makes me more aware of it, so when I do my food shop I will try and introduce some of those foods a little bit more often (B2)

Awareness / front of mind - I don’t know if it’s just because we spoke about it and that you reminded me of how I like [pulses], or having an understanding of that is actually better for a variety of different things and that makes you want to eat it more really (B2)

Awareness – Yeah, I think having the interview was a bit of a kick, it made me feel not guilty but more aware, … So, the interview, it has given me another little push (B19)

Awareness - I think it’s changed in the way that I have a more conscious, a bit like what I just said but I’ll say it again, but I have been more conscious about the other options that I have, so instead of just blindly having what I normally have I’ve been thinking oh I could put some beans on this instead, and I’ve been having baked beans quite a lot, and I know that’s not the same but I have been having them a lot more (B7)

Awareness - I guess now I feel more aware that I am actively choosing to eat pulses whereas before I thought they’re just in my diet anyway and I’m having them but now I’m thinking, oh yeah, I’m having pulses today (B10)

Awareness / consciousness raising - Your little study has made me think and challenge existing beliefs which is always a good learning curve, so I’m a little bit more aware of what I should’ve been aware of but I wasn’t (B15)

Reminder - I think our chat a few weeks ago just brought it to the forefront again and it just reminds me to bear in mind and be more mindful about what we’re eating and where it’s coming from and what it’s doing to the planet. There was even something on television the other night about palm oil so it’s good to be reminded, it’s good to have a reminder every now and then I think (B16)

Awareness - we have a lot of legumes and since the last interview we’ve had quite a lot of things like that such as kidney beans. So I would say I’m more consciously aware now how good having pulses in your diet is now. So when I’m having food that has pulses in it, I just know about it a bit more, so I’m a bit more conscious (B21)

Awareness - I’m more aware of it I guess, maybe I’m buying a few more foods that include pulses in the supermarket, for instance, just having chilli con carne a few more times because it’s got kidney beans in it (B18)

Reminder - I haven’t tried any of the recipes but I have tried some of my own, I did used to eat quite a lot of pulses but I don’t anymore so it’s kind of made me think, oh, why did I stop that? So I started making some chillis like I used to (B6)

Reminder - I think it just reminded me that actually there’s these chillis with so many beans in and vegetables and they usually make me feel really good, and I did actually think about my diet after the interview and thought, well I actually don’t feel great, so maybe I could try eating some more healthy food, more veg and beans and stuff, so that’s why I went back to that recipe (B6)

Reminder - I am still eating pretty much the same except I have been eating more beans again, I don’t know if it’s because I thought I haven’t had a chilli in a while or if I’ve been genuinely influenced by that video, but I have been eating a lot more foods with beans in, I do enjoy them anyway (B6)

Reminder - I’ve been getting two tins of beans and I’ve been going to the shops. I’ve been getting butter beans and the mixed beans (B6)

Reassurance - It’s kind of consolidated my thoughts, I rate them [pulses]. I would have them again, yeah (B9)

Awareness - I kind of subconsciously found it really interesting, so I was trying to incorporate a few more into the meals and when I went shopping I went down the tinned aisle to look at the pulses out of interest and bought a few (B13)

Awareness - I guess when I go to a restaurant, I might have it in my head now when I read a menu what pulses are and if things like chickpeas and lentils and stuff comes up then I will try that in the meal and then I might think how am I going to adopt that into my diet, so if I’ve tried it in a restaurant I have liked it so that’s what could happen in the future (B18)

Awareness - I haven’t cut down the meat, or maybe I have slightly but not intentionally because we have been having vegetable curries and stuff like that, but actually maybe I haven’t. I think I have probably realised that there are other things that you can eat that isn’t meat I suppose (B20)

**Willingness**

Willingness - I would say that my goal would be to have them often. Like probably nearly every day, maybe five times a week or six times a week, because I think they can be a really good base (B7)

Willingness - I’m always up for trying new things, so I would definitely be up for trying more pulses, I like different foods in different ways (B4)

Willingness - however as a result of trying the soup I would actually be more interested in trying to do more vegetarian or vegan meals, and using more pulses and lentils in my cooking because it was good. So I will explore that further as a result of trying the soup and because it’s healthy (B5)

Willingness - I will definitely make it again or something similar, or try to investigate some other use of lentils because they are a good source of fibre and stuff and protein (B5)

Willingness - I do want to try those black bean brownies as well, I haven’t got round to it but I am genuinely really curious about them, I wanna see what it’s like (B19)

Willingness - I haven’t, but I will try. I will try and do a recipe every now and then so that when I go to Uni I know a few recipes, and they’re a good way to bulk out meals and stuff and they’re healthy (B5)

Willingness - I am definitely going to try the brownie one. I need to go to the shop on the weekend when I’m not doing anything, but I am definitely going to do it (B19)

Willingness – [I will use pulses again]. Yeah, definitely, I already have. … I am actually looking for new ways to add them into my diet (M8)

Willingness - It’s certainly made me think about it more so I might try and have a go at some new dishes. I’m also going to try cooking chickpeas differently to see if I can add them to a homemade curry (M11)

Willingness – Absolutely. … I may try a cake with them now what I have a recipe (M12)

**Trying new things**

Trying new things - Because we did that one recipe and it just tasted so good (B1)

Trying new things - No harm in trying it once. If you don’t like it, then you don’t have to do it again, if you do like it, do it again (B1)

Trying new things - After having our interview with the benefits of pulses I’m trying the couple of recipes that you gave us. The recipes have been very nice, and if you’re telling me they are even more healthier than our existing diet then that’s brilliant, we’ve learnt something, opened up new horizons and challenged us to try something new, and it was successful (B15)

Trying new things - I would say that there is all kinds of foods that you’ll enjoy, but that you don’t know that you enjoy, so it’s worth trying things, or even one step or ingredient at a time, like I do with mine. But I think it’s definitely worth, well I think there’s a lot of vegetarian and vegan food that I’ve had which is delicious (B6)

Trying new things - I think I have tried to put some more pulses in my diet and I’ve been trying a few more things, like this morning I tried having kale for breakfast, so fried kale, with tomatoes and bacon (B7)

Trying new things - Then the brownies I was just intrigued to see what they tasted like, because I have made beetroot brownies before but I’ve never made bean brownies so I was just interested to see what they tasted like, it was just an opportunity to try a new recipe (B12)

Challenge - I think it’s quite fun. I think it’s quite fun adding a bit of a challenge to what we’re eating. (B20)

Trying new things - you suggested some recipes and that was like a challenge to do, so we went out and bought some stuff, I didn’t think it looked overly appetising, but we cooked with them, and we tried them out and stuff, and I feel like I like them a lot! (B20)

Discovery - However I think it’s a constant moving process so now I have tried some stuff, like I didn’t know cannellini beans were just baked beans without the baked bean juice. So now I have discovered a whole new range of cooking I’m going to try and get into it (B5)

Trying new things - the cake was the most unusual, the most delicious, and also the most fun to make (M1)

Trying new things - I don’t think anything [would prevent/deter me from eating pulses], because I’m always experimenting. Until I saw the recipe cards I never realised I could add them to cakes and biscuits (M6)

Trying new things - Trying them out, cooking something and its really nice. I am trying to do at least two meat free days and I, actually find I am doing more than two. My son has recently moved back in and he loves to cook. We have been experimenting together (M8)

Trying new things - I made the white bean vanilla cake, … because I have never seen pulses in a cake before and wondered what it would be like (M9)

**Small changes**

Small changes - but we are going to start doing a meatless meal a week (B1)

Small changes - For me I think it’s hard to change my whole diet. I’m, …, I tried to change my diet by one ingredient at a time. I won’t start trying to change my diet all at once (B6)

Small changes - So, whenever I have seen them or tried them it’s been an interesting experience but it’s not the main meal and it’s not the main ingredient, and I need a lot more to my dish to accompany pulses (B18)

Small changes – So, we are going to give it a go but it hasn’t completely converted me. But, I think it will be a slowly, slowly thing and I probably will includes some more of the beans in my weekly shop, so then I can include them in things like a casserole or something like that or something to thicken up a casserole rather than use what I do usually use (B11)

Small changes - Yeah a little bit, like I said, in the last three weeks I’ve had 21 meals and two of them have had pulses in it so that’s quite a good increase (B9)

Small changes - I feel like I haven’t committed fully, I haven’t changed drastically, but I have moderately changed the way I am eating, slowly (B7)

Small changes - so she (the dietitian on the video) just did it really slowly, didn’t tell anyone and nobody noticed, and then it was just normal, and that sort of made me think, oh it’s doable, I suppose (B20)

Small changes - we have tried, I think we could probably try more, but we have tried and we have done a few different recipes, the whole sweet and savoury stuff, so that was good (B20)

Small changes - I tried to adopt different things into my diet like I said at the start, and increase the pulses that I’m already eating (B18)

Small changes - I don’t think we are completely converted, but it would be good to have more pulses in your diet, and I would perhaps now look for dishes that include that. And I know it said three times a week but we might just manage once a week or even once a fortnight but we would definitely try and include it in the diary for sure (B15)

Small changes - I suppose to be fair, [we haven’t changed our diet], not a great extent, I think we are still doing very much the same as always, but there’s just been an addition of the recipes you sent over, so rather than bake a Victoria sponge we did the brownies. Rather than opening a can of Waitrose soup we had the soup recipe, so that’s changed a little bit (B15)

Small changes - minimally. I’ve taken the executive decision to get some of those beans. … I mean I’ve had those bean things twice, and I thought they’re pretty nice in the chilli (B9)

Small changes - I have been buying like I said about two cans every week now, and that’s been like three weeks, so I feel like that’s change my diet slightly (B6)

Small changes - yeah just increasing the ones I know really, like garden beans and kidney beans, and just increasing those in the types of foods that I already eat (B18)

Small changes - I would definitely say it is easy changes, and there is easy substitutions and add-ons to recipes that we already do. I actually really like the soup recipe and adding the beans to that because it made the soup a lot thicker and it felt richer and felt more filling without really changing anything, so just adding in those beans, and I think that something I can adapt into other soups, because we eat soups a lot for lunch so we could just add beans into anything and blend it up and it would be easier to make and not have to buy soup (B12)

**Enjoyment**

Liking - I liked that this had peppers in it and then I thought if I found a tomato soup that I enjoy making this would be great, and it was really easy to make and really quick to make and it tasted delicious (B10)

Positive experiences - We tried the brownies, they were chocolate brownies so nothing can be that bad, but they were … I think even you said to have them with cream for pudding and they are quite delicious then, and then we also had the tomato soup, and that was really nice. I really like that soup actually and it was really filling and flavoursome (B20)

Enjoyment - The soup recipe was delicious, absolutely delicious, so much so that I think will add that to our staple making and keep it in a big pot and ladle it out for a few lunches during cold winter days, it was delicious (B15)

Enjoyment – the cake was … the most delicious (M1)

Enjoyment - I thought it looked quite appealing and because I like mushrooms (M3)

Liking - I love chickpeas and make them when my sister comes over. Green and red lentils are OK and I will eat them with my son (M7)

**Sensory properties**

Texture - The lentils gave you something to chew on which was very important otherwise it will just be like soup. So we are looking to try and do one a week (B1)

Texture - Well when I was prepping the food, I was thinking it doesn’t look very thick, it just looks like a soup, which was a bit off putting. But when actually eating it, it was fine (B1)

Taste – Yeah, really nice but you definitely need the spices, they helped massively (B1)

Taste - I think that was down to the spices, I don’t know what the lentils added to the taste (B1)

Taste - One of them is this Iranian stew, it’s sweet potato cut into chunks with some Iranian spices, and again like your soup you make this great big pot of it and it’s delicious (B15)

Taste – [I would be deterred] if they tasted bad really. Obviously they’re pretty small so if you put them in a big meal you won’t notice them too much, but that would be the only thing … if they tasted really rotten (B19)

Appearance - well have you ever seen a pulse? They don’t exactly look thrilling do they (B5)

Flavour - I would do recipes which it wouldn’t really change the flavour of, like with the soup I would’ve added cream instead of the beans but it still tasted creamy (B14)

Sensory - I kind of understand it is things like beans and lentils and chickpeas. I just see them as they have to be small, natural, and taste horrible (B19)

Flavour – To improve it, maybe having some extra flavour in the pulse mix such as vanilla (M1)

Taste - It’s one that I particularly like as I like the taste of lentils on their own, so I make this one quite a lot (M4)

Taste / texture - I actually thought they were quite tasty, but I would probably tweak the recipe a bit. … they were a little bit dry (M3)

Appearance / Taste - I made the chickpea cookies. … I guess they just looked quite tasty (M2)

Texture - I have had kidney beans out of a tin, but I think the others might be kind of mushy though, so I haven’t tried them (M4)

Taste - I like lentils particularly. I love the taste of them on their own (M4)

Taste / texture - They were a little bit dry and bland (M7)

Appearance - I just thought that they looked quite nice (M8)

Taste - They tasted lovely, they were very nice (M8)

Texture - I have put them in a curry before, they go soft. I don’t like them in chilli con carne, I tend to pick them out as I don’t like the texture (M10)

Texture - I’m not keen on the texture of chickpeas so I tend not to eat them unless they have been boiled until they are soft. I hate kidney beans, again because of the texture. I’m the same with baked beans. I don’t like vegetables crunchy either (M11)

**Practical Concerns**

Cost - also it’s a lot cheaper than buying meat, as meat is pretty expensive in comparison, so a bit of that as well (B1)

Cost - Also I have noticed I’m going to University in September and they’re quite cheap (B5)

Cost - I think because I have been trying out new ingredients it has made me think about how it doesn’t have to be as expensive as I think it is. For example, the packets of beans and lentils that I have been using and already had in the cupboard, and especially dry things, you don’t need much of it and the bag will last you. … even though it’s hard for me to gauge and I cook too much, that just shows you can get a lot out of it out of just a small amount. So, you can get more from a small bag of pulses than you can out of buying meat where are you have to buy a few packets. So, for example you’d have to buy like four packets of chicken, packets of chicken breast, if you are going to eat that every day for like a week, whereas I could buy one bag of lentils and that would last me a few weeks (B7)

Cost - Also they were a lot cheaper than I thought they would be so I might try using them again. … They would also make a cheap meal (M2)

Cost - It actually works out cheaper and tastier to make a meal from scratch. Instead of opening a jar, a tin of tomatoes and adding lentils gives you a much better base for a tasty meal (M8)

Cost - I would say it’s been a bit more expensive. We are trying different recipes and stuff because they do work, even the things that sound weird like the avocadoes and bean brownies. They do work and you can make it to something that turns out to be nice (B20)

Waste / storage - The dry stuff because it lasts longer (B7)

Ease - just as easy to cook. Put it with a load of spices that you would’ve put with the meat anyway. So yeah, it’s quite good really (B1)

Effort – yeah, it’s not really a problem if you put your mind to it, but I can imagine a lot of people would be like, oh, I don’t know how to do it, I’ll just go and buy chicken instead (B1)

Ease – It was really easy. You just put it in a saucepan and blend it all up (B5)

Ease / quick - It was really easy to make and really quick to make (B10)

Time - it does take a long time to cook but my mum said what I could’ve done is just put the lentils to soak overnight from the day before and they would’ve been softer so they would’ve cooked a bit quicker, because it did take two hours to cook from dry (B1)

Ease - It’s just like adding pulses to soups and stuff. It’s just an easy thing to do especially as we already make our own soups (B13)

Ease - Like in every meal, the doing it is quite easy (M1)

Convenience - I always thought they had to be soaked before and didn’t realise they could be bought in cans (M2)

Ease - Not particularly easy to be fair, especially if you have to soak them overnight (M4)

Convenience - Just the soaking process as its long and drawn out but I like them, so it doesn’t put me off really (M4)

Ease / convenience – [I use] the green and red [lentils] because they don’t have to be soaked overnight. … I boil them, as it’s quick and easy. I also keep them and reheat them as I use them a lot (M4)

Ease / convenience - I find it relatively easy because I buy them in cans. That way I can add them to any dish quickly (M3)

Ease / convenience - I wouldn’t [eat pulses] on a regular basis, but if I did it would just be to heat up some beans from a tin (M5)

Ease / convenience - They were also really quick and easy to make. … Surprisingly easy, I had never really used them before but since this study I have found that I am using them more and more (M8)

Easy - I find it easier than most other dishes I cook, they can be added to all sorts (M6)

Easy – [I didn’t eat them], not before, but I do now, I’m converted! I have been throwing them into curries and fajitas (M8)

Ease / convenience - For a stew its really easy because I can just buy some lentils ready to go in a can from the supermarket and add them. They are also good to throw into a curry but I don’t really use them for anything else (M11)

Ease - It’s quite easy to add them to stews but otherwise you have to soak them the night before and I always forget (M7)

Ease / convenience - This is a faster and quicker way to add one of your 5-a-day to meals. They are also so much quicker now that you can buy ready made ones in cans, so you don’t have to soak them like before (M8)

Ease / convenience – I suppose now I see that you can buy them in a can, you could add chickpeas to a curry or maybe a casserole (M9)

Ease / convenience – I’d normally just throw them into stews and casseroles because its easy. If they weren’t available to buy in a can I don’t think I would bother because I don’t have the time to soak them (M11)

Ease - Get the tinned ones than the dry ones because they’re a bit more awkward (B14)

Effort – to be honest the soup was quite a big task to do for what it is, so I went against that (B18)

Effort – yeah, they (dried pulses) are a lot more effort, you have to know what you want for dinner the next night (B14)

Availability - I suppose having them available, because we went from a zero base with pulses and we had to buy some lentils and we had to buy some black beans. I suppose that’s the first step, actually getting the pulses into your cupboard and into your larder. Because you’ve always got a tin of baked beans and soup and you’ve always got a packet of spaghetti and a pasta mix but we didn’t have red lentils or black beans, so it was going onto the shopping list and adding on a pack of lentils, so I guess it’s the thought process of having them available so if you want to make your soup or your brownies they are there (B15)

Availability - So there is only a little [shop] across the road so if I was to go there sometimes they don’t have all the pulses in there. They do usually have black beans and stuff but some of the pulses like kidney beans and chickpeas they don’t sometimes have, which is a bit annoying and that means you have to go to a bigger supermarket, but I think that’s the only kind of thing I have thought about (B13)

Availability - remembering to order them. It’s not in my general food shop. I’m not saying I buy baked beans every week, but it’s not in my general … ‘have I got enough of that or have I got enough flour or enough herbs’ so it’s getting into that mindset. It’s just remembering to order it so when you are doing the casserole you can chuck them in, or some beans, or whatever so they are there and available. … I think I will, like I say, I think I will try and add it to my regular shopping list, and if it’s in the house then I think I will use it (B11)

Availability - we have never had legumes in our cupboards and my mum said I cooked with them, but after we talked about them I really like them, and my dad really likes them so we’ve made sure they end up on the shopping list and we’ve gone and got some more once we’ve ran out (B20)

Availability - I knew that there was a white bean range that I don’t think I realised how easy it was to get quite a few different types and I never really thought about that (B14)

Availability - I think I will be adding them to my shopping list, so we will have them in the house so then they will get used in different things, rather than thinking let’s stick in a tin of tomatoes in, it might be okay, let’s put in half a tin of tomatoes and half a tin of lentils or beans or something (B11)

Availability - we have bought quite a few which means we will be trying some more (B20)

Availability - it is more widely available than I thought, for some of the pulses and stuff, even though I had bought them before I just hadn’t realised that there was different sort of, normal bean aisle and then the international section seems to have better numbers for example (B14)

Availability - I always have them in the cupboard though (M7)

Storage - I guess the other side of it is, quite a lot of beans you can get in tins so you can buy lots of them and put them in the cupboard and they’re not really going to run out by a sell-by-date very quickly, which is a good thing. Whereas lots of fruits and vegetables we’ll have to use up quite quickly, like in a week, but with pulses they’re just in a tin so you can use them whenever you need to (B13)

Time - it’s just easier to have ready meals out the cupboard or whatever, so that’s a big thing how much time it takes, if you have a busy life and stuff. There’s just too much going on in my day to think about what to put in my meal (B19)

Time - Definitely, when I have more time I intend to try one of the recipe cards (M5)

Time - I think we have gotten more creative at the moment anyway because there’s nothing else to do, but after the first interview we put some brown and white beans on our shopping list and we’ve made dishes with both of those, and when we went shopping this week we had a lot more put on the list, so that’s been good (B20)

Versatile - they can be made into a lot of different things, so you can pick the right mill for you (B1)

Versatile - I think they are a lot more versatile than I realised (B7)

Versatility - Another reason is that I didn’t ever think about adding them to cakes and biscuits, I just thought they could be added to main meals (M2)

Versatile - Personally, I believe they can be added to anything! (M3)

Versatile - I eat it (lentil dish) all the time and just change what I have with it. Like change the meat or have fish. Maybe I could variate it with different lentils or something instead (M4)

Versatile - I cook them with anything, I love the taste. They can bulk out a meal or replace meat or fish (M4)

Versatile - To be honest I like it as it is, I sometimes add some chicken, but I could add some more vegetables to it instead (M6)

**Benefits**

Health benefits - they are very healthy for you (B1)

Health benefits - I tried the soup. I like soup, and it’s just a healthier alternative to a tin of Heinz, and I thought, why not, I’ll give it a go. It’s just a healthy soup, isn’t it, and it tastes alright so probably the health benefits may have lead me to want to make it (B5)

Health benefits - if they are becoming more popular because of health benefits reasons then yeah, definitely (B2)

Health benefits - I think it’s just because it’s healthy for your body, it’s a lot healthier way of getting protein which is just better for you so that would be my main reason for eating more (B4)

Health benefits - I understand that although that I don’t like them very much, they do have a lot of health benefits, and you don’t have to eat very much, I believe it was something like 2 cups a week is what they recommended, spread throughout your diet, obviously you don’t have to eat them all at once, but that would be a good benchmark and good amount to have, just because they are very healthy and they can also reduce things like breast cancer, obviously it’s all cancers but I think they mentioned breast cancer in particular in the video (B8)

Health benefits - I do want to try and keep introducing pulses into my diet if I can because I now realise that they also have health benefits (M2)

Health benefits - Yes, I do [eat pulses]. … I know that they are really good for you (M3)

Health benefits – [I eat pulses for] bowel health (M5)

Nutritional benefits - I think it’s just a good source of protein that isn’t purely chicken to be honest. I feel like I don’t eat enough vegetables as well, and I feel like a bit of a carnivore at the moment so I thought adding a few more beans would vary my diet a bit more (B6)

Nutritional benefits - It was very nice and an easy way to increase overall fibre content of the diet (M5)

Filling - I think that’s another really positive point and benefit about them, they do really feel you up and the meals do seem to be more filling (B20)

Filling - I think it’s a really really good way to make a meal like a hearty meal I suppose, and at the moment that doesn’t really bother me too much, I suppose because I am living at home and my mum does the shopping, but when I do all my own cooking and do all my own meals, that would be a great addition to now know about because I can make a meal so much better, and like I said earlier it can fill you up so I can imagine myself getting a bit sick of beans on toast otherwise (B20)

Filling - They are also good for bulking out a meal and I find them filling (M4)

Bulking - partly to bulk out a vegetable dish, give it some more substance (M1)

Bulking - I think you could try adding them to curries or soups to bulk them out a bit (M2)

Variety - probably the interest, as it makes a change (B7)

Variety - the one time I did adopt some more into my diet I quite enjoyed it. It was quite nice to have a bit of variation from what I usually eat, so healthy variation is always positive as well (B2)

Well being - It makes me feel better about myself to because I’m trying to do something positive and I’m trying to change my diet in a positive way (B7)

Well-being - Actually you know there is a health benefit to this, then you feel slightly more virtuous when you do eat them (B11)

Multiple benefits - they are a good source of protein and fibre, they are a very sustainable source, although you could argue if you grew loads of them then they might not be sustainable. They didn’t taste bad, well, the ones that I tried, they were alright. I would use them in my cooking, because, yeah they are a good source of fibre and protein and they’re healthy for you (B5)

Multiple benefits - They are quite good for a few different reasons. One of them being health but also from a cost point of you, you can stick in a can of beans or lentils and that reasonably small amount of money can bulk up any food stuff that you’ve got (B11)

Multiple benefits - I realised they’ve got a lot of protein in them there’s lots of different types of pulses as well, which is quite nice because you can have a bit of a variety, and they’re really healthy and they’re not that expensive (B4)

Multiple Benefits - They are cheap, tasty, and always good for bulking out a meal. Plus I had it drummed into me by mum how good they are for you (M3).

Mulitple benefits - I eat some just to add to the health benefits really, a bit of roughage for the diet and it bulks out a stew (M11)

Multiple benefits - Yeah, I use [this recipe] most weeks, not every week though, because it’s quick, easy and healthy. I get my greens, pulses and protein all in one meal (M12)

Multiple benefits - I also love lentils because they are tasty, good for you and healthy. … Definitely because I enjoy them, not only are they full of taste and nutritious but it’s something different from the normal mundane cooking and I never feel over-full or bloated after eating a lentil dish (M6)

Multiple benefits - It’s a dish that I like to have in winter as its nice and filling. Its also a good way of getting some veg into your diet (M11)

Benefit alternative - there’s gotta be a reason for it. There’s got to be something in my diet which I think is not doing any good for me and I need to swap this for something else and then I would look into something that was a good alternative (B18)

Benefits alternative - The benefits are, I imagine, adding something positive to my diet already. By adding something positive, I guess, that might mean taking something negative away, so if the pulses are pushing in somewhere, it’s got to squeeze something out the other side, so I would hope the squeeze out the other side would be something not so good for me (B15)

Benefits alternative - I did a healthier Mexican recipe so I used beans instead of meat (B16)

Benefits alternative - But actually some foods that are vegetarian like pulses are very high in protein, and they’re probably better for you as well because of all the other things, because especially red meat, isn’t really that good for you, is it? (B17)

Benefits alternative - probably it will mean that I do cut out some meat out of my diet (B20)

Benefits alternative - They are also much more healthy than using meat. Since this I have been having more and more meat free days and using pulses instead (M8)

Benefits alternative - Or as a replacement for meat. They are also a good source of protein, something healthy (M1)

Benefits alternative - They are also useful for replacing meat in some dishes for a change (M8)

Benefits alternative - I think they could be used instead of vegetables occasionally but not instead of meat because I like meat (M10)

Benefits amounts - we are probably not eating enough maybe, to notice any real difference, I don’t think (B20)

**Knowledge**

Knowledge - I did know about them, but I forgot what they were called, and now thanks to you, you have reminded me what they are and what they do, so yeah now I’ve got a better understanding (B1)

Knowledge - More knowledge, more awareness of the variety and the amount that’s out there, and really, I suppose, that if you put in white beans or black beans into a search engine at any local supermarket it doesn’t go, we haven’t got any, it says here’s the range. So that surprised me, so there was a choice (B11)

Knowledge - But I would say since our last interview I have definitely consciously thought about what pulses I am having whether that’s lentils or beans, but just actually realising there are so many benefits and they are not just an add-on. So, before it was just like, oh, I’ll chuck them in as well and now I’m consciously thinking I’ll have them because they’re better for us and there is better protein and better health benefits (B12)

Knowledge - if pulses need to be in everyone’s diet we need to have a certain amount of pulses, then the supermarkets need to make that clearer, on the news or documentaries or something. It’s adopting something that I really don’t know much about (B18)

Knowledge - I probably need to do a little bit more research but the ones that I do know, I am increasing the quantity of that in my food. So, I think it just needs a bit more exploration into - do you have a pulse and how could I maybe try this? (B18)

Knowledge - I actually had them quite a lot in my diet, but I didn’t know they were so good for you (B21)

Knowledge - I suppose it probably is doing the interview, but that’s because I probably didn’t really know anything about them I suppose, like I said before I only really eat what I know, but having talked about them, I’m totally for incorporating them further into my diet (B20)

Knowledge - I found it really interesting about the different facts about pulses, it made me want to try them, because I kind of learnt things that I didn’t know before and I just thought, oh, let’s see what we can do with them (B13)

Knowledge – Well, now I kind of know what they are and I know that they are easy to incorporate or can be easy to incorporate into meals, and the health benefits (B13)

Knowledge - What would encourage me? it would be a reason of why I should be using them, why its offering better nutrition (M1)

Knowledge - Now I’m more aware of the health benefits, it would be a good idea to have more in my diet (M11)

Knowledge - I understand they are healthy for me and media health reports say it’s a good idea to include them in your diet (M12)

**Cooking Ideas and Confidence**

Cooking knowledge - I think it’s just down to the knowledge of the nice food I can make with pulses really. I know there’s a few people that I’ve had their food from beans before and it’s been really nice (B6)

Cooking knowledge - The thing is with cooking with pulses, you do need to add lots of things to them like spices and sauces and things. So if you’re not used to doing that and cooking from scratch and adding things to a meal, you might not know how to do that, know how to make it more exciting to eat (B7)

Cooking knowledge - I guess I probably hadn’t thought about shoving a tin of pulses into my soup and then blending my soup because I don’t know how keen I would be to have just beans floating around my soup, I think that would be a bit weird, but blending them made a really nice texture so it probably has changed my soup making strategies (B10)

Cooking knowledge - What would encourage me? Secondly a bit more knowledge or exposure to recipes and dishes (M1)

Cooking knowledge - I don’t have much knowledge on where I should and could be using them. I think that’s the only thing that would put me off, probably the knowledge, the exposure, how to use and where to use them. Not a distaste or anything. I just don’t know (M1)

Cooking knowledge - Having to look up a recipe rather than just making something I know which is the easier option, especially trying to fit this into a busy lifestyle (M5)

Cooking ideas - I don’t think I really have any barriers … maybe finding recipes, but I’m sure there is a recipe book out there, I just haven’t looked for it (B1)

Cooking ideas - The only barrier being I don’t know recipes, but I could just go on my phone and search for one and there it is straightaway (B1)

Cooking ideas - There are more recipes out there to do with meat, maybe give it some time or be creative. You know, you might come up with something exciting to do with lentils (B1)

Cooking knowledge / ideas - It’s just having the knowledge of the different dishes that could accommodate them I guess. But I guess if you had that knowledge you wouldn’t necessarily think that they are boring. But if you think, oh, they can only go in a couple of things you might think they are dull (B8)

Cooking ideas - I feel like I have a better understanding of what they are and the huge variety of pulses there are, and the ways that you can use them. Like they’re not just something you can use in a chilli, you can do lots of things with them, like making the soup, for example, and the brownies. So, there are more options. (B7)

Cooking ideas - I think I would struggle with the variety of meals you could make. I only know a handful of recipes so I would end up eating the same thing all the time (M1)

Cooking ideas / alternatives - I thought maybe they could be put into a sauce and served with pasta. It would make a change from real meatballs and my mum would love that because she is a vegetarian so is always looking for alternative meals (M3)

Cooking ideas - Apart from curries or stews I don’t know, because I don’t know many recipes (M7)

Cooking knowledge - I would need to find some more recipes to know what things I could add them to (M7)

Cooking knowledge - I have no idea how to cook them or what recipes I could put them in (M9)

Cooking knowledge - My lack of knowledge mostly. If I could find some more recipes of things I like, then I wouldn’t mind buying them (M10)

Cooking ideas - Knowing how delicious it was in the soup, I might try adding pulses into things I wouldn’t of normally put pulses in. I’m not saying this is going as far as my cereal because I think that is too far, but blending it into my soup or even into a pasta sauce, it would give a really nice texture and previously I probably wouldn’t of put a pulse in it but now I will (B10)

Cooking ideas - With the brownies I guess it’s just opened up my eyes to different ways of cooking stuff (B12)

Cooking ideas - I used to use them just to fill the gap as an add-on, something like kidney beans in the chilli or just things like that as an add-on rather than making the meal around the pulse. But since the interview I have definitely realised how much the health benefits and how much I should be having, it’s definitely encouraged me to use more recipes that had beans and pulses in, and the diversity of things like the black beans in the brownies it’s not just a savoury thing, it can be a sweet and it can be in something like a soup and you can blend it up to make a different consistency (B12)

Cooking knowledge - I think people just don’t know what to do with them, I think more and more there are recipes coming out and you can add spices and stuff, but you wouldn’t just eat a chickpea on its own, you have to add it with spices and if you’re unfamiliar with doing your own spices then they probably are pretty bland. You can probably get packet mixes for them like a fajita recipe, so all the spices mixed together, and maybe that would encourage people to eat them I don’t know, but I think they are pretty bland on their own, but then chicken is pretty bland on its own, if you don’t add anything to it. So just knowing how to make them more exciting and tasty (B14)

Cooking ideas - I think it would just be finding new things to make, that would maybe be the only barrier because I can see so many ways to put beans into a recipe. And I know there are loads and loads of recipes, but I don’t know them, so I would have to find them and make them (B20)

Cooking ideas - I’m not the best with trying to be creative with food, so it’s just figuring out ways of incorporating different things into it and making it enjoyable (B19)

Cooking knowledge - It just shows that having knowledge is wonderful, all these cooking shows just show that you just need a little bit of knowledge and then it’s great (B15)

Cooking ideas - The instructions also helped, as I have never thought of making a cake with pulses before (M1)

Cooking ideas - I don’t know many recipes with lentils though (M7)

Cooking ideas – [I’d eat more if] I had some more recipes of things I do like to eat (M9)

Cooking ideas - I don’t really eat that many pulses and I don’t know many recipes so unless you were cooking a specific dish, I wouldn’t think it would be that easy. … I just don’t know what to do with them (M10)

Cooking ideas – I’m sure you could add them to a lot of things, but I don’t know any recipes. I was surprised by your recipe card that they could be added to cakes (M12)

Cooking skills - It was alright, it was a bit herbie, but I could just put less in next time. I suppose I could’ve planned it a bit more, it was a bit lumpy, I think under there is just my cooking skills. I have never made soup before in my life, but yeah, overall good and I would eat it, well I did eat it, and I’ve got still a whole tub of it left for the rest of the week probably. So, I will definitely eat it again (B5)

Cooking confidence - I suppose my beliefs now are that I am more willing to try things that include pulses, but it’s just making sure they are there and you are comfortable cooking them. Do you fry them? do you put them in water? it’s just knowing what to do with them (B15)

Cooking confidence - I probably need to use scales and look up how much is in, well I think it’s quite difficult with dry pulses to see how much that is, so it can turn out to be quite a lot and you end up eating more than you think you are because it’s not what you’re used to. It’s not the same as having a piece of meat and a piece of this on a piece of that. It’s tricky to know how much is in, like a handful of lentils (B7)

Cooking ingredients - But I did find the recipe requires a lot more different ingredients which I wouldn’t usually have in the house. I got round some of that, so if you google anything you can find an alternative for anything so I managed that. But for half a teaspoon of this or a quarter of a teaspoon of that would I buy that ingredient? Probably not (B11)

Purchasing Knowledge - If I was shopping for myself I would now know where to go for the pulses, and if I wanted to make a vegetarian curry or a black bean curry I could just go and pick up some black beans and I would now know where to go, so there’s a bit of a gain in knowledge in that for sure (B15)

Easy / Readily available information - an easy reference or pamphlet which explains where you can go for XY and Z would be quite handy for folk like me and I could put it in my recipe cupboard, and if I wanted to do something I could flag it out and use it (B15)

**Incorporation**

Incorporation - I would consider putting more things like lentils and beans in things, especially in things like casseroles (B11)

Incorporation - I think the main thing I have included is more pulses into food or I have thought about it a bit more and I have kind of been a bit like, oh, this week I could put more, I’m having veggie chilli tomorrow so I can put more pulses or kidney beans in that, so it’s a bit like that (B13)

Incorporation - we’ve had conversations where we have made dinner and we say, oh, those beans would’ve gone nice in this or something like that, so I think going forward that’s quite a good change (B20)

Incorporation - I have had black beans before and I did like them before, but I didn’t really consider them as something separate (B9)

Incorporation - I was having chilli anyway and I thought it would go in it and it would work. I just sort of chucked them in and they were done (B9)

Incorporation – [I cook them] straight from a can as I’m always on the go. I add them to soups, stews, salads, curries, bolognaise almost everything (M3)

Incorporation – [I would] throw them in a stew (M7)

Incorporation - You could put them in a stew, my mum used to do that a lot, so I wouldn’t even know I was eating them, but I don’t know any other recipes (M10)

**Cooking Solutions**

Solutions - They’re probably quite bland in terms of flavours, but realistically every flavour is bland, but you can make it into something (B2)

Solutions - I think because everyone thinks healthy food is boring. But I don’t think it is if you do it right, and if you get the balance right (B3)

Solutions - It’s the lack of knowledge on how to do things with that sort of food. I think if that was more available and slightly easier to use, I think, people would use it more probably. … So, your ability to cook it in different ways which are more enjoyable really (B4)

Solutions - I think I definitely will be [trying to include more pulses in my diet], because I will get bored of the chilli soon and then I’ll think what else can I make to sort of supplement this, and I think I’d still like to go down the pulse route (B6)

Solutions - So generally it did seem like there was a lot of benefits and not really a lot of downsides. Other than the taste, because I don’t really like them that much. I could probably live with that in certain dishes though (B8)

Solutions - I think maybe it’s switching what I buy every week in the supermarket and swapping a few things around (B18)

Solutions - I could have also added some rosemary to the lentils, I have done that before, and it comes up really nice. … Sometimes I add a bit of rosemary, garlic, or ginger to them for a change (M4)

Solutions - I really like mushrooms and thought I would try lentils mixed together with them as I am not keen on the taste of lentils on their own (M7).

Solutions - You could add an onion, and some more seasoning like thyme. I like thyme or cayenne pepper. I might also fry them instead of baking them in the oven (M7)

Solutions - I have never heard of pulses in a biscuit before and I thought it might disguise the taste (M10)

Solutions - I am not keen on the texture of chickpeas, but I love Indian Dhansak curries and I know it’s made with them, so maybe it’s the way they are cooked that I do like them. I could try cooking them a different way (M11)

Solutions - I also only put lentils in if I can disguise the taste (M12)

Solutions - Taste [is a barrier] when they’re used in recipes that I dislike. So, if I put it in something I like, I’m happy to add them (M12)

**Risk**

Reliable / risk - The main barriers is not knowing if I’m going to like it or not, or not knowing if it’s gonna fill me up and I’m going to the shop once a week and I’m not going daily so going once a week means I need to have that week‘s worth of food ready. It needs to be tasty, it needs to fill me up and it needs to be something I know how to do quick (B18)

Reliable / risk - Seeing if I like it because I only go to a supermarket once a week and I have things in the fridge which I know are tasty and I know are good, so sometimes if it’s a risk for me to buy something and then not like it, I am then disappointed (B18)

Reliable / risk - I don’t really need stuff that is new and different and I’m unsure if I’m going to like it, because I’m just working so hard and I need stuff I can rely on. So that’s kind of the big reason really. It’s almost like I’m scared to dip in and try something new and I just don’t wanna be disappointed at lunchtime and then have to think I have to eat something else and then that takes away from my work schedule (B18)

Reliable / risk - For me it’s just a risk or scared feeling that I’m not gonna like that and then having to buy something and make something on top, and it’s inconvenient (B18)

Familiarity - I do have a barrier of accepting foods that I really don’t know, …, I think it’s a risk accepting new recipes and understanding how that might work into my routine (B21)

Familiarity - So, I’m playing it quite safe at the moment because I know what I like, I’ll then buy more of it (B21)

**Preconceptions**

Preconceptions - I was surprised I liked it, I thought that I might not like it. But yeah, it was good, had those sneaky little vegetables in there which was good. But yeah, I would do it again (B5)

Preconceptions - Even the soup was so much more filling, and I know soup doesn’t normally have meat in it, but I think it’s just so much more substance than what I would’ve imagined for a meal not having meat in it (B20)

Preconception / value – I’d probably say getting those seeds and eating them in that way is more doable than I thought it was, like buying those cans of kidney beans, for example, or lentils, isn’t that expensive and does it make big meals? It’s not a waste of 70p where you’re going to chuck something in and it’s not going to make any difference (B20)

Expectations - Other than that I guess I wasn't the most highly motivated to try them as I know I don't particularly like the flavour, so I attribute that to not remembering to buy the ingredients (B8)

Expectations - because it was tomato soup and I was expecting it to be a bit bland but I think having the roasted peppers in it gave a little kick to it, which was really nice. So, I didn’t expect to hate it but because it was tomato soup I wasn’t expected to be excited by it (B10)

Expectations / Preconceptions - The fact that they are not that sweet really that should be a positive rather than negative, because we don’t need any more sugar in our diet, it’s just coming after the chocolate brownie after the oasis café. It was a bit different so I should’ve thought of it differently from a brownie (B11)

Preconceptions - Yeah we put some beans in a casserole, and that was absolutely fine. I find beans or pulses in savoury [dishes] in my mind more normal, than in some thing that I perceive should be sweet. So, that might be an easier sell (B11)

Preconceptions - making a chocolate brownie you never ever think you’re going to use black beans, you would expect to use something more cake like, so it was quite an eye-opener that something like that could substitute and it was tasty and it was a little bit of a different taste but very similar to a chocolate flour-made brownie. So, it was a big eye-opener really (B15)

Preconceptions / knowledge - I also thought they would be boring I suppose, I don’t know, I don’t think people know what they are or about them, so they kind of seem like a vegetable but they’re not really a vegetable, I don’t think I really knew what they were, let alone how to make them interesting I suppose. You don’t even need to make them interesting you can just use them and incorporate them (B20)

Preconceptions - I think I was surprised it was creamier than… was it replacing cream? because I was trying to work out what would normally be in it instead of the beans. … I did the soup, I didn’t look at the price of it compared but it’s probably cheaper and it was easy to just get it from the supermarket, you don’t have to go down to the market to get beans. I don’t know how sustainable the pack I got was, but it’s obviously still better for the environment than other things (B14)

Preconceptions – yeah, I was pleasantly surprised, if someone told me to taste it I probably wouldn’t have realised that there was beans in it (B14)

Preconceptions - I suppose I thought it was gonna be a little bit harder, I don’t know why I thought it would be harder, it is just blending it up (B5)

Expectations - I wasn’t expecting it to be as good as it was (M1)

Preconceptions - I would probably use it to impress someone, or if they had an intolerance to certain ingredients, or to surprise someone as it’s quite unusual (M1)

Preconceptions – It was much easier [to make] than I thought it would be, I don’t really use pulses in everyday cooking normally, so this was good. … They were tastier than I thought they would be. … They tasted better than I expected them to (M2)

Expectations - I didn’t realise they would taste so good, they actually taste nice, and they are very cheap to buy (M8)

Preconceptions - I didn’t think it would be that easy because I thought you had to always soak them first. It wasn’t till I went shopping that I realised you can buy them in cans (M9)

Preconceptions - Until I saw the recipe cards I never thought they could be added to a cake!! (M11)

**Forgetting / Laziness**

Laziness - It’s not that I didn’t like the sound of them, I’m just lazy (B2)

Laziness - Just laziness if I’m honest and I forgot (B4)

Forgetting - I didn’t get a chance. It would slip my mind or I wouldn't remember while I'm at the shops. I don't go very often, so within the timeframe it didn't come up (B8)

Forgetting - I don’t do the food shop and I forgot to ask for the ingredients but I do want to do the brownie in the future (B3)

Forgetting - I slightly forgot about making the soup until yesterday. However, from yesterday I’m definitely going to investigate more lentil and pulse uses (B5)

Laziness – [I might eat more if I] get someone else to cook for me (M12)

**Habit**

Habit - probably just habit, it’s just not an ingrained habit to have pulses in my diet. Because I am used to more traditional options like we just talked about, like wheat and processed things, it is difficult to go completely over (B7)

Habit - I find it difficult to eat less, especially when I tried a lentil dish, I over eat I find it difficult to figure out how much to have (B7)

Culture - I don’t know I think it might be because they haven’t been exposed to a lot of legumes and pulses before and I think it depends on their culture or certain types of culture that don’t incorporate it and it hasn’t been passed down, then it wouldn’t really appeal to them (B21)

Upbringing - I wasn’t really brought up to eat them so tended to eat other things instead (M2).

Upbringing – [Using pulses is] difficult, but only because I wasn’t taught how to do this growing up (M5)

Habit – Habit mainly, I tend to stick to the same foods most of the time (M2)

Habit - The problem is I mostly only cook for myself and probably wouldn’t think about it as I tend to eat the same things each week. My mum makes casseroles when I go home but I eat pizzas and pasta and don’t usually try new things (M9)

Habit - As I don’t really buy chickpeas, I probably wouldn’t think to get them. They aren’t something I tend to pick up when I go shopping (M10)

Habit - I tend to eat the same meals most weeks (M10)

Habit – What would deter me? I suppose time and habit. We tend to cook the same things most of the time (M11)

**Influence of Others**

Influence of others - I think if I did the food shop I would be, but I don’t do the food shop so I don’t really have a say (B3)

Influence of others - I don’t really do too much of the shopping (B2)

Influence of others - I think for me it’s largely that I don’t control what is being bought in my house, so that’s probably one (B5)

Influence of others - And when I was making meals, I was making for more than one person and they weren't the kind of dishes I’d do for people, as I'm not familiar with them at all (B8)

Influence of others - because you encouraged me to, that’s probably the reason, which I have tried it. If you hadn’t sent it to me probably not. I don’t think I would’ve gone out my way to find a recipe which had beans in it (B11)

Influence of others - I needed to cook for a family of four so it was just a good opportunity to do it, whereas if I just did the soup or the avocado brownie they’d have been more just snacks, or the soup is just a starter or a lunch, so I went for a full meal (B1)

Influence of others - What would encourage me to eat them more? Probably if my flat mates were to eat them too. We do tend to share the cooking and if they used them more that would probably make me use them more too (M2)

Influence of others – Yes, I do [eat pulses]. My mum’s a vegetarian (M3)

Influence of others - My husband doesn’t really like them, so I don’t bother making them just for myself (M7)

Influence of others - My son loves them and sometimes he will cook them for me (M7)

Influence of others - I tend to cook the same things for me and my husband (M7)

Influence of others - I don’t really use them regularly as I wouldn’t just cook them for myself (M7)

Influence of others – What would encourage me? If I was eating with someone else and they ate them (M9)

Influence of others - My dad and step-mum tend to do most of the cooking too, so I eat what they eat (M10)

**Compatibility**

Compatibility / suitability - I haven’t done much with pulses, but I have done other things, and I have tried to incorporate other things more. And the dishes that we have been making haven’t really had a course to have the pulses in. So if it works then I wouldn’t be opposed for sure (B8)

Compatibility - I don’t need to increase it anymore as I already eat plenty. I have them most days (M4)

**Missing Nutrients**

Missing nutrients - There are probably some things in meat that you do get but you don’t get in pulses, but I haven’t really looked into it. I’m not really that worried about it because I’m only gonna do one meal a week veggie anyway (B1)

Missing out - I don’t really want to cut down on my meat intake, I suppose that’s a barrier (B20)

**Awareness, but**

Awareness, but - I guess I was thinking about with the beans and stuff, because I tried the recipe for the soup and when I was looking down the aisle with the lentils and stuff there is quite a range especially in the international section. So I was thinking I should buy more, but did I buy more at the time? no, but I would like to look at recipes and stuff, so not much has changed since the last interview in terms of what I’ve actually done, but I do definitely think about it more (B14)

Awareness, but - I have definitely thought about what I’m doing with my diet since the first interview, I can’t be honest and say I have done much about it (B19)

Awareness, but - Did it change what I bought? Probably not. Apart from buying a few more beans and stuff like that for the recipes (B14)

Awareness, but - I did eat them once but other than that it’s just more of a thought and an idea than actually going out and changing at the moment (B2)

Awareness, but - It’s made me think a bit more about beans, because for example one of my friends girlfriends are vegetarian so they don’t eat a lot of meat, he loves meat but he’s quite happy to eat quite a lot of veggie food because he does eat a lot of beans these days, and I do quite enjoy his cooking and I thought do you know what, not that I have acted on it. But I have been thinking how could I incorporate beans or maybe vegetables with high protein into my diet rather than just relying on chicken all the time. So I had thought about it, yeah, but I wouldn’t say that I have definitely acted on it. I may have acted on it slightly by buying beans, but I haven’t started exploring many recipes yet (B6)

Awareness, but - yeah definitely I will try it, I will always try something before I just disregard it and say that I don’t like it. But it would just be a case of having it when it came up and thinking, oh, shall we give this a go, but off the cusp I wouldn’t really be trying it. So, I would definitely give it a go at one point, but I don’t know if that would be any time soon. But I wouldn’t really have any reason to not give it a go as such, unless I know for a fact it’s something that I don’t like (B8)

Awareness, but - I think I might [make this recipe again] if I had chickpeas in the cupboard, but I wouldn’t go out specially to buy them (M10)
